# Super-resolved trajectory-derived nanoclustering analysis using spatiotemporal indexing

**DOI:** 10.1038/s41467-023-38866-y

**Published:** 2023-06-08

**Authors:** Tristan P. Wallis, Anmin Jiang, Kyle Young, Huiyi Hou, Kye Kudo, Alex J. McCann, Nela Durisic, Merja Joensuu, Dietmar Oelz, Hien Nguyen, Rachel S. Gormal, Frédéric A. Meunier

**Affiliations:** 1 Clem Jones Centre for Ageing Dementia Research, Queensland Brain Institute, The University of Queensland, Brisbane, QLD 4072 Australia; 2 School of Mathematics and Physics, The University of Queensland, Brisbane, QLD 4072 Australia; 3 Queensland Brain Institute, The University of Queensland, Brisbane, QLD 4072 Australia; 4 School of Biomedical Sciences, The University of Queensland, Brisbane, QLD 4072 Australia; 5 Present Address: Australian Institute for Bioengineering and Nanotechnology, The University of Queensland, Brisbane, QLD 4072 Australia

**Keywords:** Image processing, Functional clustering, Super-resolution microscopy

## Abstract

Single-molecule localization microscopy techniques are emerging as vital tools to unravel the nanoscale world of living cells by understanding the spatiotemporal organization of protein clusters at the nanometer scale. Current analyses define spatial nanoclusters based on detections but neglect important temporal information such as cluster lifetime and recurrence in “hotspots” on the plasma membrane. Spatial indexing is widely used in video games to detect interactions between moving geometric objects. Here, we use the R-tree spatial indexing algorithm to determine the overlap of the bounding boxes of individual molecular trajectories to establish membership in nanoclusters. Extending the spatial indexing into the time dimension allows the resolution of spatial nanoclusters into multiple spatiotemporal clusters. Using spatiotemporal indexing, we found that syntaxin1a and Munc18-1 molecules transiently cluster in hotspots, offering insights into the dynamics of neuroexocytosis. Nanoscale spatiotemporal indexing clustering (NASTIC) has been implemented as a free and open-source Python graphic user interface.

## Abstract (web summary)

Existing single-molecule localization microscopy analyses overlook important temporal information in living cells. Here, the authors report nanoscale spatiotemporal indexing clustering (NASTIC), which leverages a video game algorithm to fast-track the investigation of the complex temporal dynamics of protein clustering.

## Introduction

**Fig. 1:**
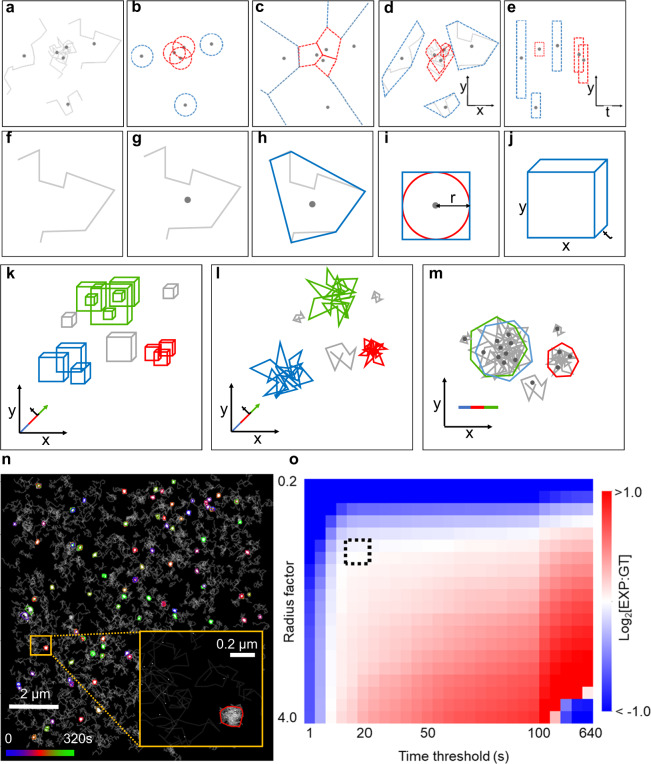
Schematic representation of clustering algorithms as applied to molecular trajectories. **a** Molecular trajectory data, with each trajectory’s spatial centroid indicated with a dot. **b** DBSCAN. Multiple molecular centroids present within a defined radius (red circles) are considered clustered. The most effective radius (**ε**) and the minimum number of centroids within it (MinPts) are determined empirically. **c** Voronoï tessellation. Tiles are drawn around each centroid such that the distance from any point within the tile is closer to its centroid than to any other centroid. Molecular centroids with tile areas less than an empirically determined threshold (red) are considered clustered. **d** Spatial indexing. Clustered molecules are determined by overlapping 2D bounding regions (red), defining the spatial extent of each molecular trajectory. **e** Spatiotemporal indexing. This panel represents the data in panel (**d**) rotated 90° around its *y*-axis to highlight the temporal component of each centroid. Each trajectory bounding region is assigned a user-defined “thickness” in the time dimension. Overlapping 3D bounding regions represent spatiotemporally clustered molecules. **f** Molecular trajectory composed of individual detections. **g** Spatiotemporal centroid representing the trajectory’s average position in space and time. **h** Convex hull (blue) defining the spatial extent of the trajectory. **i** Simplified 2D spatial bounding box (blue square) based on the approximate radius (r) of the convex hull (red circle). **j** 3D spatiotemporal bounding box of user-defined “thickness” in the time dimension. **k** R-tree spatiotemporal index of all trajectory bounding boxes. Discrete clusters of overlapping bounding boxes are indicated in color, and unclustered boxes are in gray. **l** 3D clusters of trajectories associated with overlapping bounding boxes. **m** 2D representation of clustered trajectories. Colored polygons represent the spatial convex hull of all detections comprising each of the clustered trajectories. Clusters are colored according to the averaged detection time of their component trajectories, allowing the assignment of overlapping clusters (green and blue) occupying the same spatial extent at different times. **n** Nanoscale spatiotemporal indexing clustering (NASTIC) of simulated trajectory data described in *“Optimum parameters for spatiotemporal clustering”* using *r* = 1.2, *t* = 20 s. Cluster boundaries represent the extent of the detections associated with clustered trajectories and are colored according to the average detection time. The inset displays a zoomed view of a single cluster against a background of unclustered trajectories, with trajectory centroids indicated with a dot. **o** Heatmap of averaged metrics (cluster number, cluster radius, trajectories per cluster and the number of clustered trajectories, see Supplementary Fig. 3). Each pixel represents the average log_2_ ratio of the experimental observed (EXP) value for a given *r/t* pair to the ground truth (GT). Pale regions indicate *r/t* pairs which return cluster metrics close to the ground truth. The approximate inflection point where the pale line transitions to horizontal is indicated with a dotted box. Source data are provided as a Source Data file.

**Fig. 2:**
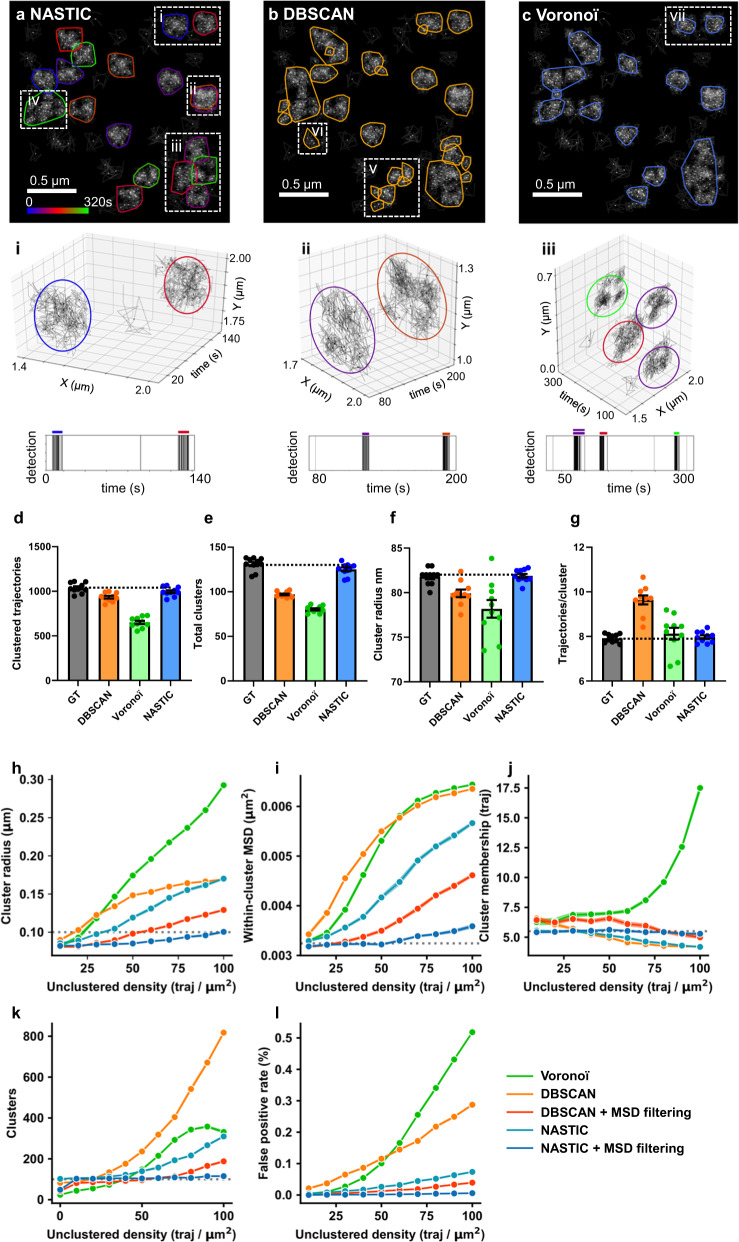
Comparison of clustering algorithms. **a**–**c** Resolution of spatiotemporal clusters in simulated data as described in *“Comparison of clustering algorithms using simulated trajectory data”*. **a** Clustering using NASTIC using *r* = 1.2, *t* = 20 s. Insets highlight different classes of clustering: (**i**) distinct clusters resolved in space and time; (**ii**) spatially overlapping clusters resolved in time; (**iii**) clusters with a degree of spatial and temporal overlap; (**iv**) clusters which overlap in space and time. 3D (*x*, *y*, *t*) projections of highlighted clusters (**i**–**iii**) and the associated detection times (lower panels) demonstrate distinct temporal clustering. **b** DBSCAN spatial clustering using **ε** = 0.055 μm and MinPts = 3. **c** Voronoï tessellation spatial clustering. Trajectories with an associated Voronoï tile area <0.004 μm^2^ were considered clustered. In all analyses, a cluster is defined as three or more proximal centroids. **d**–**g** Comparison of cluster metrics returned by NASTIC, DBSCAN and Voronoï tessellation from synthetic data simulating 10 acquisitions as described in *“Comparison of clustering algorithms using simulated trajectory data”*. **d** Total trajectories in clusters, **e** Total unique clusters, **f** Average cluster radius (nm) and **g** Average trajectories in a cluster. Black bars represent the ground truth (GT) in the simulated data, colored bars represent the metrics returned by DBSCAN (ε = 0.05 μm, MinPts = 3, orange), Voronoï tessellation (tile threshold 0.01 μm^2^, green) and NASTIC (*r* = 1.2, *t* = 20 s, blue). Error bars show the standard error of the mean (SEM) across 10 datasets. The dotted black line shows the average value in the input synthetic data. **h**–**l** Comparison of the ability of different algorithms to return metrics matching the ground truth as the density of unclustered background detection increases in a synthetic dataset. Source data are provided as a Source Data file.

In recent years, great advances have been made in our understanding of cellular molecular dynamics through the emergence of super-resolution microscopy, and in particular, a suite of technologies collectively referred to as single-molecule localization microscopy (SMLM)^1^. When applied in live cells, this approach allows the detection and tracking of individual molecules at the nanometer scale, far below the diffraction limit of light, thereby opening the way to understanding the nanoscale world of living cells^2^. Single-molecule tracking of proteins at the plasma membrane has revealed that membrane-associated proteins can congregate into functional assemblies called nanoclusters. SMLM has allowed the characterization of molecular nanoclustering of synaptic receptors and their functions^3,4^. The formation of these nanoclusters can be studied to allow dynamic characterization of single receptors and their effectors confined into these discrete areas of the plasma membrane^5–13^. These studies seek to define metrics such as mobility, nanocluster size, lifetime, molecular membership and density and establish how they correlate with changing experimental conditions. The key to such investigations is a robust analytical pipeline for determining which of the hundreds/thousands of molecular detections (Fig. 1a) acquired in a typical single-particle tracking experiment are confined into nanoclusters. To date, algorithms for nanocluster determination have largely relied on one of two fundamental principles: (1) density/proximity algorithms such as DBSCAN (density-based spatial clustering of applications with noise)^14^ which determines clustering based on whether the number of molecular detections within a determined radius exceeds a user-defined threshold (Fig. 1b), and (2) segmentation-based algorithms such as Voronoï tessellation^15,16^, which define minimum area tiles around each molecular detection and assign clustered detections based on a user-defined tile area threshold (Fig. 1c). More recently, computer vision has also been used to determine clustering based on algorithmic identification of features in SMLM data^17^. However, these techniques have been mostly developed for fixed cell data, and they are therefore lacking the temporal aspect, which is critical when considering live-cell single-particle tracking datasets. More recent approaches using Bayesian cluster analysis of live-cell data have been described^18^, which provides cluster information over time on a population level but not at the individual cluster level.

In this study, we propose an alternative approach to examining molecular clustering in live-cell SMLM data, which is based upon two primary assertions: (1) in live-cell data, the molecular trajectory is the indivisible unit for each tracked molecule, not the individual molecular detections comprising the trajectory, and (2) trajectories which spatially and temporally overlap with other trajectories are more likely to represent molecules that are confined into functional nanoclusters. We consider that the ability to simultaneously derive information about the spatial and temporal interactions of molecular trajectories can provide valuable insights into functional dynamic protein interactions on the cell membrane.

Accordingly, we investigated the use of bounding regions encompassing the extent of each molecular trajectory over the lifetime of its observation. Overlapping bounding regions represent an increased likelihood that their underlying molecular trajectories constitute members of a nanomolecular spatial cluster (Fig. 1d). Determining whether complex geometric shapes overlap is computationally intensive, particularly if there are large numbers of these shapes. This can be overcome using spatial indexing, which allows rapid querying of a database of the shapes’ rectangular bounding boxes. Over the last few decades, a number of approaches to spatial indexing, such as Quad-tree^19^ and R-tree^20^, have been implemented, variations of which have found wide use in mapping and database management, as well as in video games, where they allow highly optimized and accurate spatiotemporal detection of interactions between objects such as bullets and combatants^21^. Considering that spatiotemporal interaction is highly relevant in defining the nanoclustering of molecules, we used the R-tree spatial indexing algorithm to determine the overlap of the bounding regions of hundreds/thousands of trajectories. R-tree databases can have an arbitrary number of dimensions, so in addition to the *x*/*y* spatial extent, we included an additional time extent (trajectory detection time ± user-defined time window). This approach allowed the determination of single-molecule trajectory overlap in both space and time (Fig. 1e), and introduced a temporal component into the clustering metrics, allowing us to determine apparent cluster lifetime, rate of cluster formation and “hotspots” - discrete areas of the plasma membrane with recurrent molecular clustering. Using both synthetic and experimentally derived SMLM data, we compared nanoscale spatiotemporal indexing clustering, hereafter referred to as NASTIC, with DBSCAN and Voronoï tessellation clustering and demonstrated effective and efficient spatiotemporal resolution of molecular clusters across a wide range of detection densities. We mathematically validated our approach using vector autoregression analysis to confirm that clustered trajectories identified by NASTIC were indeed spatially confined. The nanoscale spatiotemporal metrics returned by NASTIC enable insights into the dynamics of proteins on the plasma membrane.

## Results

### NASTIC workflow

We used a Python implementation of the R-tree spatial index (https://pypi.org/project/Rtree/) incorporated into a framework utilizing the common Python modules SciPy, SciKit Learn, Numpy, Seaborn and Matplotlib. Spatial indexing requires the use of rectangular bounding boxes rather than irregular bounding regions (convex hull—the boundary enclosing a series of points such that there are no concavities) encompassing a typical trajectory (Supplementary Fig. 1). The closer the convex hull is to circularity, the closer the idealized bounding box area will be to the area of the bounding box which encompasses the entire extent of the trajectory. Conversely, the bounding box encompassing a more elliptical convex hull may lead to a significant overestimation of the area occupied by the trajectory (Supplementary Fig. 1). For the purposes of spatiotemporal indexing, we therefore used an idealized square bounding box based on the approximate radius of the convex hull (Fig. 1f–i). The square bounding box was extended into the time dimension by allocating a user-defined time “thickness” that encompassed the duration of the tracked molecule (Fig. 1j). The resulting 3D (*x*, *y*, *t*) bounding box was indexed into a 3-dimensional R-tree database (Fig. 1k). Bounding boxes in the R-tree database were sequentially queried to determine whether they overlapped with any other bounding boxes (Fig. 1k, colored boxes). The trajectories associated with these clusters of overlapping bounding boxes were then determined (Fig. 1l). A convex hull of the spatial extent of all detections associated with the clustered trajectories was used to define each cluster area and colored according to the average acquisition time of the component trajectories (Fig. 1m). An important feature of NASTIC is that identification of temporally distinct clusters occupying the same spatial extent is an intrinsic feature of the analysis and does not require post hoc processing. Querying the R-tree and defining spatiotemporal clusters is rapid, taking <1 s for ~5000 trajectories on a standard i7 laptop.

### Optimum parameters for spatiotemporal clustering

All clustering algorithms require user-defined parameters to account for the density and distribution of SMLM data. Low-density data decrease the likelihood of adjacent detections, which can be considered clustered, while high-density data, conversely, increase the likelihood of spurious clustering. Similarly, data in which detections are concentrated into multiple discrete areas within an acquisition window (e.g., axonal or dendritic neuronal projections in highly polarized neuronal cells) pose analytical challenges compared to data acquired from the plasma membrane of a single larger and flatter cell (e.g., a neurosecretory pheochromocytoma PC12 cell). Currently adopted spatial clustering algorithms require optimized user-defined parameters to best represent the molecular clustering dynamics of the data. As an example, consider an SMLM dataset for which individual trajectory centroids have been established. For DBSCAN, the radius (ε) around each molecular centroid in which to scan for neighboring centroids (Fig. 1b) and the minimum number of centroids within this radius (MinPts) to be considered a cluster must be determined empirically. For Voronoï tessellation (Fig. 1c), the threshold tile size below which a centroid is considered clustered must also be established against the average tile size or by using randomly distributed coordinates of equivalent density^15,16^. In all cases, the need to empirically determine optimized parameters for clustering exposes the potential for operator bias. In theory, NASTIC should assign trajectories into clusters based purely upon overlapping spatiotemporal boundaries without the need for user input. In practice, two parameters are required:

1. A bounding box radius factor (*r*). The nature of R-tree indexing requires a rectangular bounding box (also known as a minimum bounding region) for each trajectory. This simplified square bounding box (Fig. 1i) only encompasses the full extent of the trajectory if its original convex hull (Fig. 1h) is perfectly circular (Supplementary Fig. 1). Applying a radius factor *r* > 1 is necessary to create a bounding box more representative of the full extent of the trajectory.
2. A time window (*t*), in seconds. This defines the temporal “thickness” of the bounding box (Fig. 1j). For example, for *t* = 20, each trajectory is queried for other spatially overlapping bounding boxes 10 s prior and 10 s after its temporal centroid (Supplementary Fig. 1g–i). Greater values of *t* increase the likelihood that spatially overlapping bounding boxes will also overlap in time to generate a discrete temporal cluster. A value of *t* equal to twice the total acquisition time will, in effect, return purely spatial clustering, given that no trajectory can be considered temporally distinct from any other within this large time window.

We simulated trajectory data with controlled density and spatiotemporal distribution for preliminary evaluation of each clustering algorithm’s ability to designate clusters matching the ground truth inherent in the input data. We first generated a small dataset approximating the scale and density of a typical super-resolution acquisition at 50 Hz over 320 s. These data consisted of 50 randomly distributed trajectories within 4 μm^2^ and 0–320 s, interspersed with 20 discrete clusters, where each cluster contained 20 trajectories within a 0.1 μm radius and a 10 s time window. Each trajectory was a random walk with 8–30 segments of length <0.1 μm with 20 ms between segments. A dataset was chosen that exhibited clusters of various types: (1) clusters discrete in space and time; (2) clusters that overlapped in space and time; (3) clusters that partially overlapped in space and time; and (4) clusters which overlapped in space but not in time. From the point of view of functional nanoclustering, the latter class of clusters is particularly important as they model functional hotspots on the plasma membrane, which can repeatedly recruit molecules to a site of biological activity, such as the synaptic active zone^22–24^.

Initially, NASTIC was performed using a limited range of *r* and *t* values, representative results of which are shown in Supplementary Fig. 2. We found that *r* = 1.2 and *t* = 20 s returned spatial clusters most representative of the distribution of synthetic random walk trajectory data. Lower values of *r* and/or *t* returned multiple small spatiotemporal clusters for each of the discrete input clusters. Conversely, higher values returned fewer and larger clusters.

We expanded the exploration of the parameter space using a larger synthetic dataset consisting of 100 spatially distinct cluster regions randomly distributed on a simulated 100 μm^2^ membrane area. These data were selected to mimic routinely acquired biological single-particle tracking Photoactivated Localization Microscopy (sptPALM) data. Twenty percent of the regions constituted hotspots where 2–4 clusters occupied roughly the same spatial extent but occurred at different time points over the simulated 320 s acquisition. The final dataset consisted of 1095 trajectories in 140 spatiotemporally unique clusters with 7.82 ± 0.16 trajectories per cluster, with cluster radii of 74.86 ± 5.29 nm. The synthetic data also contained a background of 1000 randomly spatiotemporally distributed unclustered trajectories (Fig. 1n). NASTIC analyses were performed using a matrix of *r* (0.2–4.0) and *t* (1–640 s) values, and for each *r/t* pair the following metrics were evaluated: (1) number of trajectories in clusters; (2) the total number of spatiotemporally discrete clusters; (3) the number of trajectories associated with each cluster; and (4) the cluster radius. These metrics were compared against the ground truth values for the dataset and plotted as heatmaps of log_2_[observed:ground truth] for each *r/t* pair (Supplementary Fig. 3a). These plotted data reveal a complex relationship between *r*, *t* and each cluster metric. Blue and red pixels represent *r*/*t* pairs whose output was lower or higher than the ground truth, respectively. We selected lines of adjacent pale pixels highlighting the *r/t* pairs returning metrics more closely matching the ground truth. We specifically looked for the inflection point where these lines transitioned from vertical to horizontal to minimize since these represent the region of parameter space that is least subject to deviations from the ground truth in response to changes to either parameters. When the pixels were averaged across the four metrics, the resulting plot (Fig. 1o) demonstrated an inflection point around *r* = 1.2–1.4 and *t* = 15–20 s. This inflection point was also in accordance with other measures such as the Adjusted Rand Index (ARI) and Intersection over Union (IoU)^25^ (Supplementary Fig. 3b). We further investigated the applicability of these parameter values across a range of synthetic data encompassing various trajectory densities, length, and acquisition frame rates (Supplementary Fig. 4). In all subsequent NASTIC analyses, *r* = 1.2 and *t* = 20 s were used as the default parameters.

### Comparison of clustering algorithms using simulated trajectory data

Having established that NASTIC could resolve clusters in simulated data, we sought to compare spatiotemporal indexing with density-based and segmentation-based clustering on the same data. Using the smaller synthetic dataset described above, we performed a qualitative comparison of NASTIC using optimized parameters (*r* = 1.2, *t* = 20 s) against two widely used spatial clustering algorithms, DBSCAN and Voronoï tessellation. For fair comparison purposes, the clustering comparison was achieved via similar Python frameworks differing only by the commonly used Python modules implemented for clustering: SciKit Learn DBSCAN and SciPy.Spatial Voronoi. For DBSCAN, the centroids of all trajectories were analyzed using ε = 0.055 μm and MinPts = 3, which were chosen to return spatially distinct clusters of similar dimensions to the input data (radius ~0.1 μm). For Voronoï tessellation, all trajectory centroids were thresholded such that tiles with an area <0.004 μm^2^ were considered clustered. This value was empirically determined to best yield clusters reflective of the input data. Clustered tiles were grouped into discrete clusters if they shared one or more edges with other clustered tiles. In all three cases (NASTIC, DBSCAN and Voronoï), a cluster was defined as three or more proximal centroids. The spatial extent of each cluster was determined by a convex hull of all the trajectory detections associated with the cluster and the trajectories, centroids and clusters visualized by Python Matplotlib (Fig. 2a–c).

NASTIC was able to resolve the 20 clusters’ input data into 18 distinct clusters of approximately equal size consistent with the 0.1 μm random distribution radius used to generate each cluster (Fig. 2a). Observed clusters represented: (Fig. 2ai) distinct clusters resolved in space and time; (Fig. 2aii) spatially overlapping clusters resolved in time; and (Fig. 2aiii) clusters with a degree of spatial and temporal overlap. A single larger cluster (Fig. 2aiv) was also observed, which was consistent with two clusters overlapping in both time and space, accounting for the remaining two clusters in the input data. 3D (*x*, *y*, *t*) projections of the data demonstrated that spatiotemporal indexing resolved the clusters that were distinct in both space and time. Trajectories that occupied the same spatial extent (Fig. 2aii) could be resolved into discrete spatiotemporal clusters, as could trajectories with some degree of spatiotemporal overlap (Fig. 2aiii). Importantly, DBSCAN and Voronoï tessellation, neither of which can natively resolve in the temporal dimension, failed to separate these overlapping clusters, reporting overlapping and proximal (touching) spatiotemporal clusters as single spatial clusters. Further, in some cases, DBSCAN resolved areas of higher density within a spatial cluster into smaller clusters (Fig. 2bv). DBSCAN also reported areas of background “noise” as clusters (Fig. 2bvi). When compared to both NASTIC and DBSCAN, Voronoï tessellation returned slightly smaller clusters as the centroids on the edge of a cluster had Voronoï tiles larger than the 0.004 μm^2^ threshold (Fig. 2cvii).

We expanded the comparison by creating 10 randomized synthetic datasets based upon the same seed conditions as described above for our exploration of *r/t* values on clustering metrics. These datasets consisted of 100 spatially distinct cluster regions randomly distributed on a simulated 100 μm^2^ membrane area. Twenty percent of the regions constituted hotspots where 2–4 clusters occupied roughly the same spatial extent but occurred at different time points over the simulated 320 s acquisition. Each dataset thus consisted of approximately 140 spatiotemporally unique clusters of 6–10 trajectories per cluster, with cluster radii of approximately 82 nm. Clusters constituted approximately 1100 trajectories against a background of 1000 randomly spatiotemporally distributed unclustered trajectories. The 10 datasets were analyzed using NASTIC (*r* = 1.2, *t* = 20 s), DBSCAN (**ε** = 0.05 μm, MinPts = 3) and Voronoï tessellation (tile threshold 0.01 μm^2^). For all algorithms, the parameters were determined empirically to optimize returned metrics corresponding to the ground truth of the input synthetic data.

As shown in Fig. 2d–g, NASTIC consistently returned metrics most closely matching the ground truth of the simulated data. Although DBSCAN and NASTIC both reported similar numbers of trajectories within clusters, DBSCAN was unable to resolve the clusters in the hotspots and therefore reported cluster numbers closely matching the 100 input cluster regions. This also resulted in DBSCAN reporting higher average numbers of trajectories in a cluster due to spatially overlapping but temporally distinct clusters being treated as single larger spatial clusters. Voronoï tessellation consistently reported fewer clustered trajectories, and fewer and smaller clusters, with slightly more trajectories within each cluster. In our hands, both NASTIC and DBSCAN can be considered to return data reflecting the spatial clustering of the trajectories, with NASTIC natively returning more accurate data reflecting the unique spatiotemporal clustering of the trajectories. We further explored the ability of these algorithms to tolerate the increasing density of unclustered trajectories in the synthetic dataset. Depending on the metric evaluated, all algorithms deviated from the ground truth as the density increased. In order to increase robustness in high-density data, we introduced filtering based on mean square displacement (MSD) such that trajectories with MSDs larger than the mean value of the MSD for all trajectories were not indexed into the R-tree. NASTIC with MSD filtering was highly robust and returned metrics approximating the ground truth even at a very high density of unclustered trajectories (Fig. 2h–l). Our trajectory-based approach allows easy implementation of MSD filtering, which increases the robustness of NASTIC across a range of data densities. However, caution should still be applied to high particle density data from biological samples as they carry substantial risks of mistracking and ideally should be avoided at the time of acquisition. The results obtained by Voronoï tessellation clustering may reflect issues of the algorithm to accurately segment lower-density trajectory centroid information, as opposed to higher-density detection information. In any case, Voronoï tessellation could not distinguish temporally distinct clusters and would be expected, at best, to match the metrics returned by DBSCAN.

### Clustering analysis of syntaxin1a-mEos2 super-resolution imaging data

While simulated data offer the ability to precisely model trajectory density and clustering, the molecular environment within a living cell is far more varied and dynamic and represents a greater analytical challenge. We therefore next applied NASTIC, DBSCAN and Voronoï tessellation clustering to sptPALM^1,26–28^ data obtained from syntaxin1a (Sx1a) tagged with the fluorescent protein mEos2 in live neurosecretory PC12 cells (Supplementary Fig. 5). Sx1a is a member of the SNARE protein family that is located on the plasma membrane of neurons and neurosecretory cells and is involved in mediating synaptic and neurosecretory vesicle fusion^6,11,29–31^. As anticipated, all three techniques were able to detect spatial clustering of Sx1a-mEos2, with NASTIC also able to natively assign temporal information to the clusters.

**Fig. 3:**
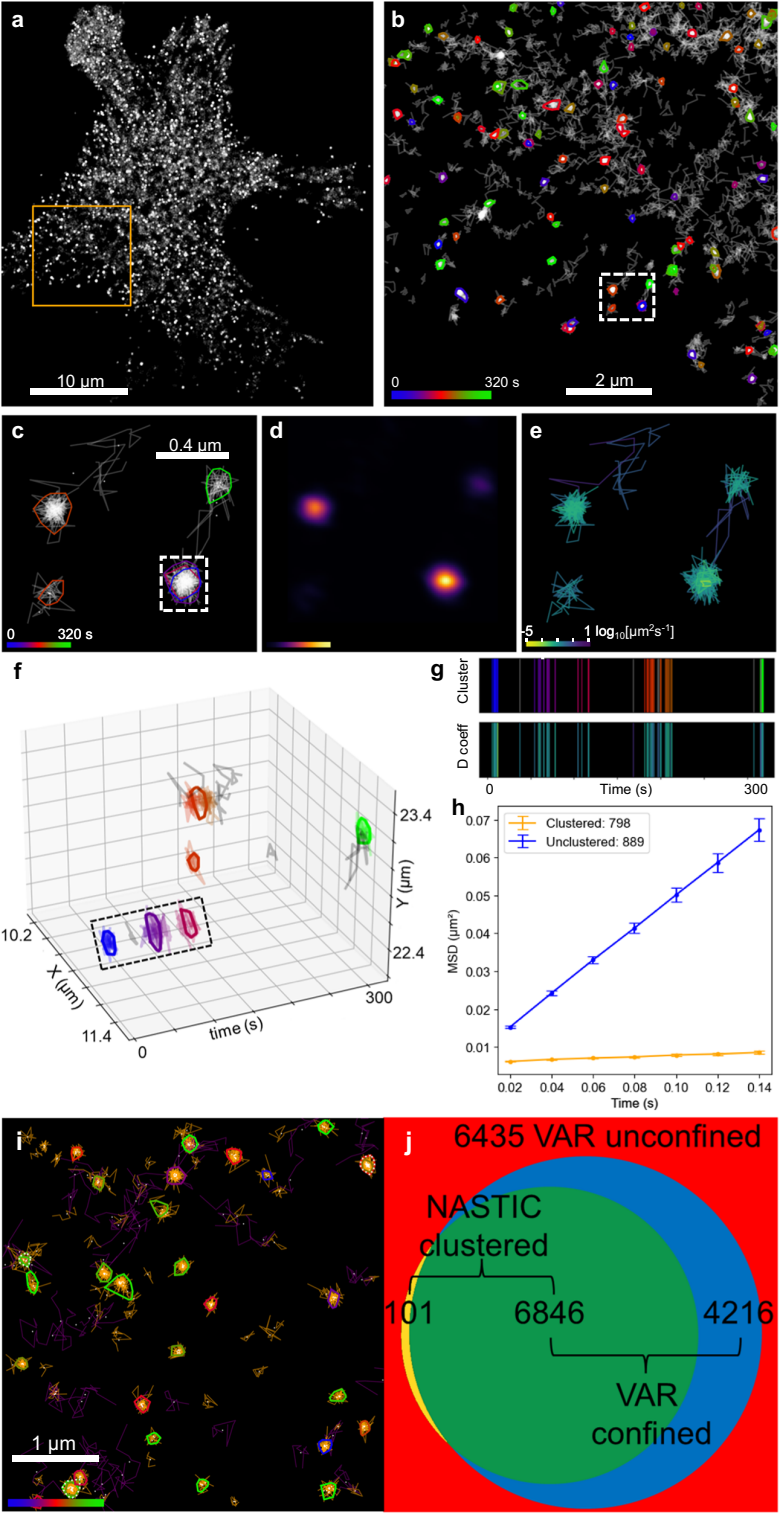
Resolution of spatiotemporal clustering in live-cell molecular trajectory data. Sx1a-mEos2 sptPALM data acquired at 50 Hz over 320 s. Clustering using NASTIC using ***r*** = 1.2, ***t*** = 20 s. **a** Raw acquisition data showing all molecular detections, with the region of interest (ROI) highlighted in yellow. **b** Spatiotemporal clustering of the selected trajectories within the ROI of (**a**), with a region highlighted with a dotted white box for enlargement in (**c**). **c** Enlargement of highlighted area in (**b**) showing individual trajectories and their centroids, with clusters highlighted and color-coded according to their time in the acquisition. The dotted box highlights a hotspot of repeated clustering. **d** 2D Kernel density estimation of the detections associated with the selected trajectories, with brighter blobs corresponding to higher density. **e** Instantaneous diffusion coefficient, with each trajectory colored according to the gradient of the first four time points in its mean square displacement (MSD). **f** 3D plot of the selected trajectories, rotated to show the temporal separation of the clusters highlighted in (**c**). **g** 1D plot of the selected trajectories where each vertical bar represents a single trajectory, colored according to its cluster status (top panel) or instantaneous diffusion coefficient (bottom panel). **h** MSD curves of clustered and unclustered trajectories from the ROI displayed in (**b**). Each point represents the average MSD of the indicated number of trajectories. Error bars indicate the standard error of the mean (SEM). **i**, **j** NASTIC clusters of Sx1a-mEos2 comprise “confined” molecules. The entire 17,598 trajectories dataset, as visualized in (**a**), was selected for analysis. **i** K-means clustering of MSD and vector autoregression metrics for each trajectory was used to assign them into confined (orange) and unconfined (purple). **j** Venn diagram showing the degree to which clustered trajectories established by NASTIC are represented by confined trajectories established using vector autoregression (VAR). Source data are provided as a Source Data file.

The 100 μm^2^ region of interest analyzed (Fig. 3a) encompassed 1687 trajectories with a minimum of eight steps, of which 798 were assigned based on spatiotemporal indexing to 105 clusters (Fig. 3b). The average number of trajectories associated with each cluster was 7.6 ± 0.563, and the average radius of each cluster was 88.396 ± 3.541 nm. On average, a cluster displayed an apparent lifetime of 16.317 ± 1.467 s. In addition to discrete spatiotemporal clusters, we identified a total of eight hotspots comprising 18 clusters (conservatively defined as those clusters whose centroids are separated by less than 0.5 of the average cluster radius) where Sx1a-mEos2 molecules appeared to be repeatedly recruited to the same region of the plasma membrane^6,12,32–35^ (Fig. 3c, f). Clusters were associated with regions of higher molecular detection density (Fig. 3d) and with regions of lower molecular instantaneous diffusion coefficient (Fig. 3e, g). As evidenced by mean square displacement (MSD) curves for unclustered and clustered trajectories (Fig. 3h), these clusters likely represent nanomolecular assemblies where Sx1a-mEos2 was constrained into lower mobility states. If these clusters were merely artifacts of randomly overlapping trajectories, the mobility of clustered and unclustered trajectories would be expected to be similar.

### Identification of loosely interacting Sx1a-mEos2 trajectories by iterative clustering

The 105 “primary” time-resolved Sx1a-mEos2 clusters identified by NASTIC above represented those whose individual trajectories overlapped within a 20 s time window. We next sought to identify and characterize any remaining “secondary” clusters representing trajectories that overlapped at any time within the 320 s acquisition window. The 889 unclustered trajectories from the primary analysis described above were therefore reanalyzed using *r* = 1.2 and *t* = 640 s (Supplementary Fig. 6a, b). A further 337 trajectories were assigned to 53 individual secondary clusters. Rather than discrete circular nanomolecular structures, these secondary clusters tended to represent unconfined trajectories whose larger bounding boxes increased the likelihood of overlap during the acquisition. This is evidenced by the MSD curve for these secondary clusters (Supplementary Fig. 6c, d), which shows less mobility difference between unclustered and secondary clustered trajectories. The generally diffuse nature of the secondary clustered trajectories strongly contrasts with the dense, compact circular nature of the time-resolved primary clusters (Supplementary Fig. 6b) and lends further support to the ability of time-resolved spatiotemporal indexing to identify true nanomolecular clustering against a significant background of higher mobility unclustered trajectories.

### Confirmation of molecular clustering using orthogonal mathematical evaluation of trajectory confinement

As shown in Fig. 3, NASTIC was clearly able to identify clusters of Sx1a-mEos2 molecules based on the spatiotemporal overlap of molecular trajectory bounding boxes. The considerably lower MSD of clustered trajectories, as determined by NASTIC (Fig. 3h), was indicative of lower mobility of clustered molecules. This apparent immobilization could stem from a combination of increased interaction with other Sx1a molecules and/or interactors and reduced local plasma membrane fluidity reflecting localized changes to phospholipid, phospholipid metabolite and other lipid composition^36,37^. While diffusion metrics such as MSD are a useful indicator of overall molecular mobility, vector autoregression (VAR)^38^ applied to trajectories can provide useful additional metrics related to the molecule’s spatial confinement based on the iterative change in position. Therefore, for each trajectory, we derived (1) the alpha coefficient of the MSD (i.e., the degree to which the molecule’s motion is confined, directed or freely diffusing), (2) the spatial area encompassed by the trajectory, (3) the norm of the VAR coefficient matrix and (4) the norm of the VAR covariance matrix, where 3 and 4 collectively specify the degree to which the position of each point in the trajectory is dependent on the preceding point and the contribution of randomness. K-means clustering of this 4-dimensional data was used to assign trajectories into two groups, one of which exhibited the mathematical hallmarks of confinement versus the less confined trajectories in the other group. These groupings were used to conditionally color the trajectories in the NASTIC cluster output, which showed that clustered trajectories almost entirely comprised those belonging to the VAR “confined” group (Fig. 3i). Across the 17,598 trajectories analyzed, 6947 were assigned into clusters using NASTIC. Of these, 6846 (98.5%) represented VAR confined. Conversely, of the 10,651 trajectories deemed unclustered by NASTIC, 6435 (60.4%) were VAR unconfined. A further 4216 VAR confined trajectories were not clustered by NASTIC, as they did not spatiotemporally overlap with other trajectories (Fig. 3j). Only a small number (101) of trajectories that were clustered by NASTIC were considered as unconfined by VAR. Taken together, these data suggest that the spatiotemporal bounding box overlap approach used by NASTIC identifies molecules whose movement is more confined as a result of being constrained within clusters.

### Statistical analysis of Sx1a-mEos2 spatiotemporal inhomogeneity

**Fig. 4:**
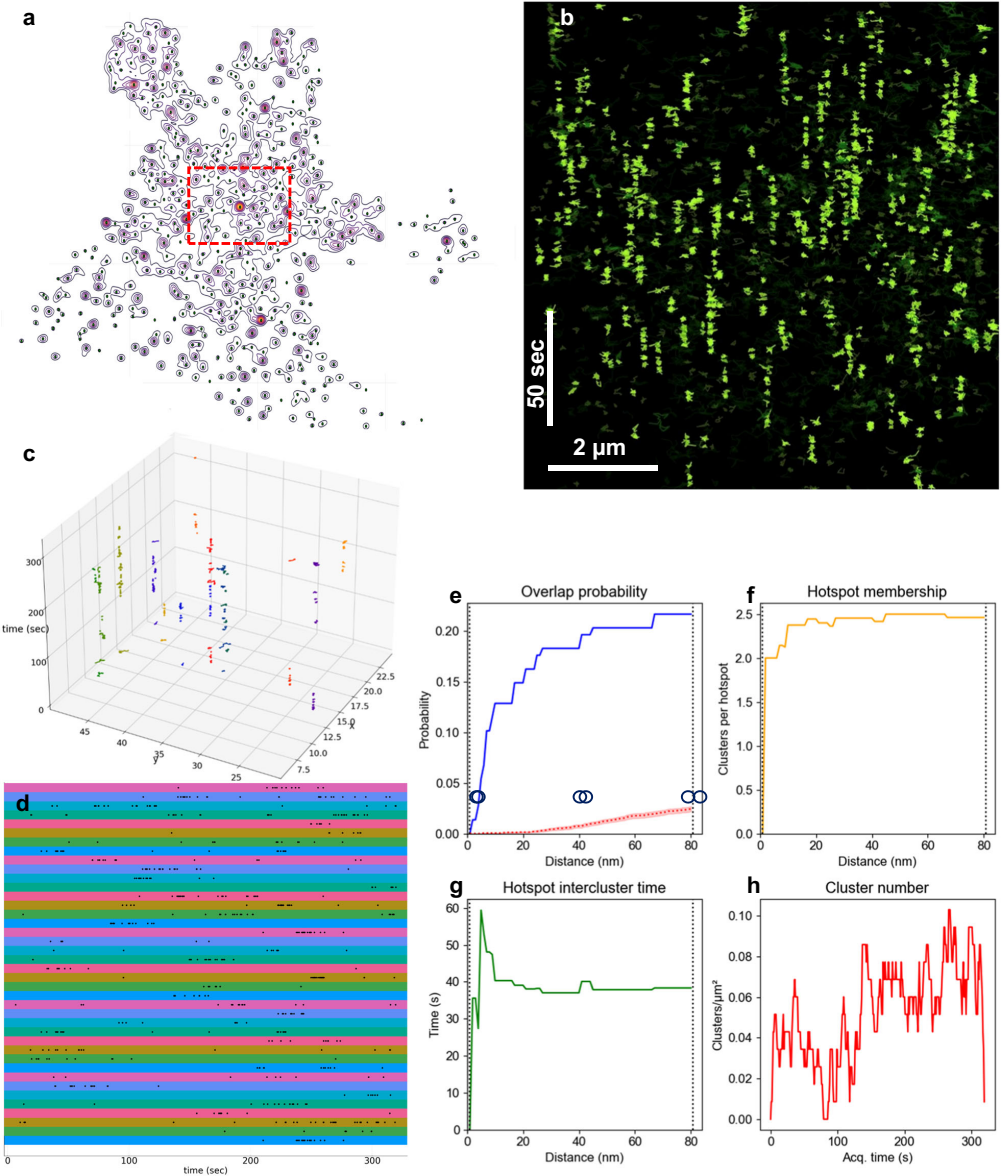
NASTIC spatiotemporal metrics. Sx1a-mEos2 sptPALM data acquired at 50 Hz over 320 s analyzed by NASTIC. **a** Contour plot of the kernel density estimator (KDE) for the spatiotemporal centroids of all 17,598 trajectories in the Sx1a-mEos2 dataset projected into the *x–y* plane. Green dots mark local maxima. **b** Orthogonal projection of Sx1a-mEos2 trajectories highlighting VAR confined trajectories (bright green) to emphasize the temporal columns of clustered trajectories. **c** Trajectories within 0.2 µm of the local maxima, as shown in (**a**), are represented as temporal vertical columns. **d** Column-wise 1D scatter plots of centroids projected onto the temporal axis for a random selection of 40 columns with significant non-uniform detections of Sx1a (*p*-value > 0.01/409 using Kolmogorov–Smirnov test for non-uniformity). **e** Probability of cluster overlap using DBSCAN of cluster centroids identified by NASTIC, **ε** = 0.001–0.083 μm (average cluster radius) and MinPts = 2. Monte Carlo simulation (*N* = 50) using 172 randomly distributed cluster centroids was used to establish the degree of random overlap of clusters of the same number and density as the experimental data. The dotted red line indicates the average overlap probability and translucent red indicates the standard error of the mean. The left and right dotted vertical lines represent 0.001 μm and 0.083 μm, respectively. At 0.001 μm, two clusters must essentially completely overlap to be considered as a hotspot, as illustrated by the overlapping circles. At 0.083 μm, two clusters are considered members of a hotspot if their edges touch, as indicated pictorially by the two touching circles. **f** Average number of clusters in a hotspot as a function of distance. **g** Average time between clusters in a hotspot as a function of distance. **h** Number of unique spatiotemporal clusters observed at 1 s intervals over the 320 s acquisition. Source data are provided as a Source Data file.

Acquisition density is a critical consideration for SMLM, particularly for live-cell imaging, where algorithms for assigning consecutive molecular detections into trajectories depend on sparse labeling to avoid mistracking. However, with lower-density data, the inherently stochastic nature of the SMLM process requires that care must be taken when interpreting any observed non-uniformity of spatiotemporal distribution, which might simply represent random “close encounters”. In this case, across a large enough dataset, spatial and temporal distribution of trajectories should trend toward uniformity. To test this hypothesis independently of NASTIC, we computed the kernel density estimator (KDE) for the spatial coordinates of all 17,598 trajectories in the Sx1a-mEos2 dataset. This was used to generate a contour plot indicating the presence of local maxima of high molecular density (Fig. 4a). A rectangular subregion (Fig. 4a, red box) containing 11,909 trajectories was selected to test for temporal distribution uniformity. For these trajectories, we conducted Kolmogorov–Smirnov (KS) tests for uniformity with respect to both the *x*-axis and *y*-axis projection of the spatiotemporal centroids of the trajectories and generated a *p*-value for each. Both the *x*-axis and *y*-axis KS tests yielded *p*-values that were lower than machine precision (<2.2E-16), demonstrating the overall non-uniformity of the temporal distribution of the trajectories.

Having earlier established that NASTIC clusters likely represent non-random confined Sx1a molecules, we sought to determine whether the observed hotspots of recurring Sx1a spatial clustering represented an underlying (potentially switchable) membrane mechanism acting to periodically spatially trap the molecules. This process would be expected to lead to strong temporal non-uniformity of molecules within hotspots. To test this hypothesis, we selected trajectories within 0.2 µm of the local maxima in the KDE contour plot of all Sx1a data, in effect deriving 409 potential hotspots, each containing at least seven trajectories. These can be represented as temporal vertical columns (Fig. 4c). KS tests for uniformity with respect to the distribution of the temporal centroids of the trajectories in each temporal column were conducted, controlling for a family-wise error rate (FWER) of 1% according to Bonferroni’s inequality^39^ such that 108 columns were identified with a *p*-value >0.01/409, indicating a highly significant degree of temporal non-uniformity. A subset of these is visualized in (Fig. 4d). These represent 108 hotspots with recurrent non-uniform detection of Sx1a within clusters.

### Characterization of spatiotemporal hotspots

Having mathematically determined that the temporal clustering of Sx1-mEos2 into hotspots empirically appeared to represent non-random events at the plasma membrane, we sought to derive metrics to characterize the recurrent formation and dissociation of these hotspots over the acquisition period. These metrics were obtained using DBSCAN on the spatial centroids of the 105 clusters identified by NASTIC, in essence treating hotspots as clusters of clusters. DBSCAN was performed using MinPts = 2 and ε values in the range 0 → average cluster radius. Overlap probability (Fig. 4e) measures the likelihood of a cluster centroid having another cluster centroid within a given distance. This is computed as 1 (unclustered centroids/total centroids). At very small values of ε, the chance of any cluster having a proximal cluster is low, as these clusters must occupy essentially the same spatial extent. Conversely, at a distance corresponding to the average cluster radius, the likelihood of a proximal cluster increases, as these clusters essentially need only to overlap slightly. To determine the degree to which cluster overlap was driven by biological distribution rather than chance, we performed a Monte Carlo simulation (*N* = 50) using 105 clusters randomly distributed over the same 100 μm^2^ analysis area (Fig. 4e, red plot). The result of this analysis demonstrates that random cluster overlap contributed little to the observed overlap. Hotspot membership (Fig. 4f) defines the average number of clusters detected in each hotspot. Intercluster time (Fig. 4g) measures the average time between clusters in each hotspot. Together these analyses show that there was an approximately 17% chance of any given Sx1A-mEos2 cluster forming a hotspot with another cluster within 41.5 nm (half the average cluster radius). The average hotspot contained ~2–3 clusters and the average time between each cluster in a hotspot was 45 s. Cluster number (Fig. 4h), measured as the number of discrete spatiotemporal clusters per μm^2^, varied over the duration of the acquisition but did not dramatically trend up or down. This is indicative of a potential “steady-state” of clustered Sx1a-mEos2 on the plasma membrane. As shown in Supplementary Fig. 7e–h, NASTIC was clearly able to identify regions of the plasma membrane (highlighted boxes) where molecules were recruited into clusters by lateral trapping over many tens of seconds. Via a user-defined time window (*t*), NASTIC could assign these multiple trajectories into discrete temporal clusters, which then allowed the additional hotspotting metrics described above. The functional significance of these hotspots is currently unknown but could represent docking/priming sites at the plasma membrane^40^.

### Using spatiotemporal indexing to define activity-dependent changes in nanoclustering dynamics

A growing body of literature has demonstrated that the spatial and temporal nanoscale organization of key proteins in the neuroexocytic pathway can change in an activity-dependent manner. These changes may reflect the functional clustering of a range of proteins required for the synaptic vesicle docking, priming, fusion and recycling at the heart of neuronal synaptic communication^41^. They may also reflect activity-dependent protein conformational changes, which alter the protein’s homo- or heterodimerization^42^. Analysis of molecular trajectory data obtained from SMLM experiments can provide insights into aggregate changes in the mobility of molecules, using metrics such as MSD as an indirect measure of the degree of their potential confinement in nanomolecular clusters. These can be further expanded using statistical techniques such as Hidden Markov Modeling (HMM^43,44^) to partition a molecular population into mobility states and transitions between them^9,45,46^, and Ripley’s K functions^10,17,47^ to derive insights into the point dispersion and cluster size. More directly, clustering analysis can reveal pertinent metrics related to the number and size of clusters, the number of molecules within clusters, and their apparent lifetimes and rates of formation. These metrics can be averaged across sufficient datasets and compared between experimental conditions, such as non-stimulated and stimulated cells, to assign statistical significance to the degree of change. As demonstrated herein, NASTIC allows significant expansion of nanocluster analysis to include metrics such as the extent of molecular clustering hotspots, the number of temporally distinct clusters within these hotspots and the time between these clusters. We sought to establish the degree to which NASTIC could generate statistically significant measures of the change in nanocluster dynamics of another key neuroexocytic priming protein, Munc18-1 tagged with mEos2, in response to stimulated exocytosis in PC12 cells. Accordingly, sptPALM data were acquired from 10 cells under unstimulated conditions and following stimulation with BaCl_2_ (2 mM). NASTIC analysis was performed on individual cells. For each cell, the metrics for each cluster were generated, together with average metrics for all clusters in the cell. Two comparison analyses were then performed: (1) the metrics for the 2610 pooled clusters detected across nine unstimulated cells were compared with those from 2588 pooled clusters observed across the nine stimulated cells, and (2) the averaged metrics from nine unstimulated cells were compared with those from nine stimulated cells (Supplementary Fig. 8). These analyses suggested that secretagogue stimulation of PC12 cells resulted in significantly smaller clusters of Munc18-1. This is in agreement with our previously published work using autocorrelation of fast Fourier transformed image data^11^ demonstrating that Munc18-1 exits the confinement of nanocluster in response to stimulation following opening and engagement of cognate Sx1A in SNARE complex formation. The rate of detection of new trajectories over the apparent lifetime of the cluster appeared higher in the stimulated cells, which might reflect activity-dependent changes to the dynamics of molecular recruitment into clusters.

### NASTIC of individual trajectory segments versus entire trajectory bounding boxes

**Fig. 5:**
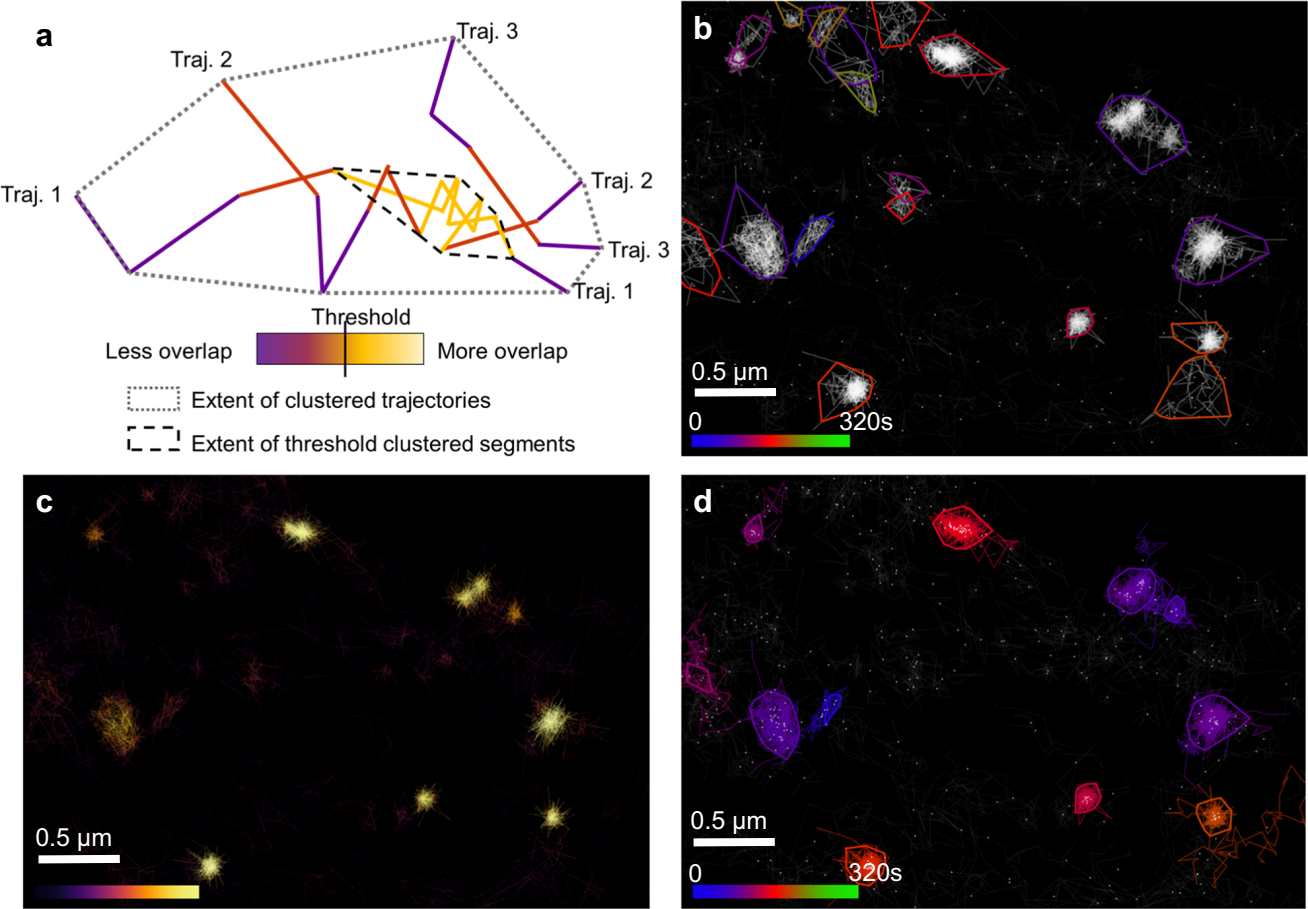
NASTIC of trajectory segments (segNASTIC). **a** Schematic representation of trajectory segment thresholding based on overlap with segments from other trajectories. **b** Sx1a-EGFP imaged by uPAINT using Atto-647-labeled anti-GFP nanobodies in PC12 cells. Spatiotemporal clusters were identified using spatiotemporal indexing of trajectory bounding boxes using ***r*** = 1.2 and ***t*** = 20 s. Each colored cluster boundary represents the convex hull of the detections belonging to all trajectories in the cluster. **c** Pseudo-density map of trajectory segment overlap, with each trajectory colored according to the number of overlaps with other trajectory segments, as determined by spatiotemporal indexing of segment bounding boxes. **d** Spatiotemporal clusters identified using thresholded segments ***t*** = 20 s. Each colored cluster represents the convex hull of detections belonging to the clustered segments. All trajectories containing clustered segments are shown in the same color as the cluster. Source data are provided as a Source Data file.

Spatiotemporal indexing of trajectory bounding boxes allows the determination of clusters of overlapping trajectories which potentially interact in space and time. However, the more precise locations within the cluster where the overlap occurs are not recovered. As schematically represented in Fig. 5a, the boundary of a NASTIC cluster represents the furthest extent of the individual detections of all the overlapping trajectories in the cluster. Within this cluster, individual segments of each trajectory (a segment is defined as the line connecting a molecular detection with its subsequent detection) will overlap with segments from other trajectories. These represent regions within the cluster where there is an increased likelihood of molecular overlap. A threshold can be determined based on the degree of segment overlap, beyond which the segments exhibiting overlap can be considered clustered. In scenarios of very high trajectory density and/or very long trajectories, determining clusters solely on overlapping trajectories (or DBSCAN/Voronoï tessellation of trajectory centroids) may lead to large clusters of low segment density (as demonstrated in Supplementary Fig. 5b), which do not truly reflect potential molecular overlaps. We therefore investigated the application of spatiotemporal indexing to the bounding boxes of each individual trajectory segment (segNASTIC) in an effort to gain more fine-grained clustering information in high trajectory density data. Each trajectory segment was assigned a bounding box comprising its *x* and *y* extent, with a time “thickness” as described above. Trajectory segment bounding boxes were indexed into a 3D R-tree, which was queried to generate lists of potentially spatiotemporally overlapping segments. From these lists, the degree of overlap of each segment with segments from other trajectories was determined. Across all segments in an acquisition, a histogram was generated showing that the majority of trajectory segments had low overlap (Supplementary Fig. 9). The inflection point of the histogram (the average segment overlap) was chosen as the automatic threshold beyond which a segment was considered as potentially clustered. Clusters of thresholded overlapping segments were derived, representing more tightly defined areas of the plasma membrane where molecules were confined. We examined the benefit of this approach using data acquired by uPAINT (universal point accumulation for imaging in nanoscale topography^48^) analysis of PC12 cells expressing Sx1a-EGFP (C-terminal tag) tracked by Atto-647-labeled anti-GFP nanobodies applied extracellularly^11^. uPAINT acquisitions generally result in a higher density of relatively longer and more diffuse trajectories when compared to sptPALM, as they use organic dyes, which are brighter and less prone to photobleaching. As demonstrated in Fig. 5b, spatiotemporal indexing of whole trajectory bounding boxes successfully identified clusters of confined trajectories in the uPAINT data, as well as clusters consisting of relatively “diffuse” trajectories. The spatiotemporal indexing of trajectory segment data was represented as a pseudo-density map of trajectory overlap (Fig. 5c), which clearly showed regions of higher potential molecular interaction. The convex hull of the detections in each group of overlapping thresholded segments was used to define a unique spatiotemporal cluster. The trajectories associated with the clustered segments were colored, which enabled us to demonstrate the disparity between the size of the segment clusters and the extent of their parent trajectories (Fig. 5d). Compared with trajectory clustering, segment clustering generally returned smaller more tightly defined clusters, and far fewer clusters of trajectories with diffuse segments.

However, this additional resolution does come at the price of the increased computational overhead of creating and querying a spatial index with 10–100 as many bounding boxes, resulting in an approximate doubling of the total analysis time when compared to NASTIC (Table 1). To compare the relative analysis times of NASTIC, segNASTIC, DBSCAN and Voronoï tessellation, we used these pipelines to analyze a dataset consisting of 4207 trajectories (a subset of the Sx1a-mEos2 data presented in Fig. 3). In our hands, DBSCAN was capable of performing the clustering of trajectory detections faster than any other methods. However, NASTIC natively delivered additional temporal clustering information that neither DBSCAN nor Voronoï tessellation could provide. Interestingly, segNASTIC, which in addition to temporal information, also provides increased spatial cluster resolution in very high-density data, has an analysis time comparable to purely spatial clustering by Voronoï tessellation.

**Table 1:** Relative clustering analysis times

ANALYSIS TIME	NASTIC	segNASTIC	DBSCAN	VORONOÏ
**Selecting detections in ROI**	1.05	1.05	1.17	1.11
**Trajectory metrics (including bounding boxes)**	6.29	3.697	2.74	2.45
**Clustering**	0.49	13.34	0.02	12.7
**Cluster metrics**	1.93	1.402	1.72	1.36
**TOTAL**	9.76	19.489	5.65	17.62

For each of the clustering pipelines, the time (s) to complete each stage of the analysis of the same dataset containing 4207 molecular trajectories is indicated. The analysis time does not include the time taken to visualize and display the clustered data.

Having demonstrated that segNASTIC returns smaller clusters representing the truly overlapping regions of each trajectory, we next compared the metrics returned from analysis of the complete Sx1a-mEos2 dataset (17,598 trajectories) by NASTIC and segNASTIC as examined in Table 1. As shown in Table 2, while both analyses returned similar overall clustering metrics, segNASTIC, as expected, returned smaller clusters. Given that different clustering algorithms use different mathematical approaches to determine molecular crossover, how does one truly define the spatial extent of a nanomolecular cluster? NASTIC uses the convex hull around all of the detections of the clustered trajectories, while segNASTIC uses the convex hull around the detections associated with overlapping trajectory segments (and thus reports smaller clusters). Beyond practical experimental concerns with data density, the choice of a given algorithm ultimately rests on its ability to detect experimentally and biologically driven changes in molecular clustering.

**Table 2:** Comparison of NASTIC and segNASTIC on a typical dataset

METRIC	NASTIC	segNASTIC
**Selection area (μm²)**	1721.44	1721.44
**Selected trajectories**	17,598	17,598
**Clustered trajectories**	6651	6857
**Unclustered trajectories**	10,947	10,754
**Total clusters**	1032	908
**Hotspots (overlap at 0.5 cluster radius)**	69	62
**Clusters in hotspots**	151	131
**Average clusters in hotspots**	2.19	2.11
**Percentage of clusters in hotspots**	14.63	14.42
**Trajectories per cluster**	6.44 ± 0.13	7.55 ± 0.21
**Apparent lifetime (s)**	11.72 ± 0.36	12.97 ± 0.42
**Avg. MSD (μm²)**	0.0066 ± 8.43E-5	0.0068 ± 9.44E-5
**Radius (nm)**	77.51 ± 0.77	66.55 ± 0.88

For each of the clustering pipelines, the metrics returned from analysis of the same Sx1a-mEos2 dataset.

### Two-molecule analysis using NASTIC

**Fig. 6:**
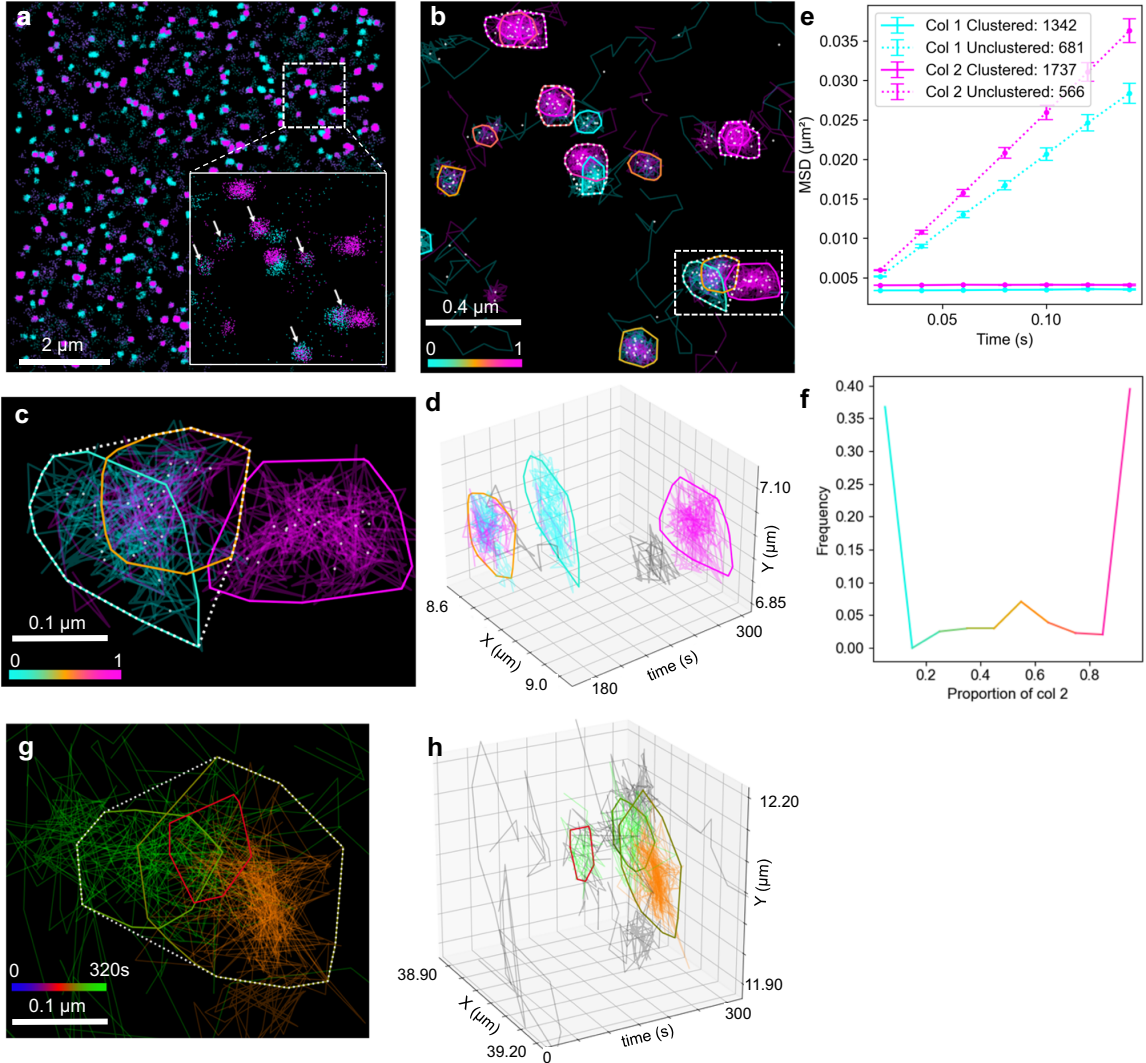
Two-color NASTIC (NASTIC2C) of simulated trajectory data. **a** Individual molecular “detections” colored according to molecule 1 (cyan) or molecule 2 (magenta). The dotted box represents the area expanded to show regions representing detections from both colors occupying the same spatial extent (arrows) and expanded in (**b**) to show clusters. **b** Spatiotemporal clusters identified by spatiotemporal indexing of combined trajectory bounding boxes using ***r*** = 1.2 and ***t*** = 20 s. Each colored cluster boundary represents the convex hull of the detections belonging to all trajectories in the cluster. Clusters are colored according to the relative proportions of component molecules, with pure cyan and pure magenta indicating clusters consisting solely of molecule 1 or molecule 2, respectively. Dotted lines represent hotspots of repeated cluster formation. The dotted white box represents an area of spatiotemporal overlap expanded in two and three dimensions in (**c**) and (**d**), respectively, to show the resolution of spatiotemporally overlapping clusters into discrete clusters with different molecular compositions**. e** Mean square displacement (MSD) curves of clustered and unclustered trajectories from each simulated dataset. Each point represents the average MSD of the indicated number of trajectories. Error bars indicate the standard error of the mean (SEM). **f** Distribution of the relative contribution of color 2 across the 436 observed spatiotemporal clusters. **g**, **h** Experimentally observed co-clustering of Munc18-1-mEos2 (green) and Syntaxin-GFP-Atto647 (orange). Source data are provided as a Source Data file.

The spatiotemporal clustering approach employed by NASTIC should readily lend itself to two-molecule analysis in which potentially interacting molecules of interest are labeled with fluorophores with different excitation/emission maxima and SMLM data simultaneously acquired at two different wavelengths^49^. We investigated this potential application using simulated trajectory data representing two-color SMLM acquisitions. Two datasets were generated, each consisting of clustered low mobility random walk trajectories against a background of unclustered higher mobility trajectories, essentially as described in Fig. 1. The datasets primarily differed in the mobility of their simulated trajectories, such that molecule 2 trajectories on average contained 10% longer trajectory segments. To simulate the potential interaction of the two different molecules, each dataset was generated using a number of shared “seed” points such that the combined datasets contained clusters consisting of both molecule types and spatial hotspots of clusters of either molecule type. Two-color NASTIC (NASTIC2C) was performed by combining the datasets and establishing spatiotemporal overlap of all trajectories as described. The type (color) information for each trajectory in the combined datasets was retained, which allowed the calculation of the relative contribution of each molecular type to the resulting clusters and was used to inform the graphic output; 436 unique spatiotemporal clusters were observed, with 52 clusters assigned to 25 hotspots. In addition to identifying clusters of each molecular type, NASTIC2C was clearly able to identify mixed clusters with varying ratios of molecule 1 and 2 (Fig. 6a–d, f) and resolve areas of spatiotemporal overlap (Fig. 6c, d).MSD curves of unclustered and clustered trajectories (Fig. 6e) further demonstrated the ability of NASTIC2C to resolve clusters of lower mobility trajectories and also reflected the ground truth mobility differences of the two synthetic datasets. To demonstrate the applicability of NASTIC2C in an experimental context, it was then used to highlight spatiotemporal co-clustering in live neurosecretory cells^31,50,51^. We reveal that, in discrete area of the plasma membrane, Munc18-1-mEos2 and Syntaxin-GFP-Atto647 co-cluster in space and time (Fig. 6g, h).

## Discussion

The increasing sophistication of SMLM research has resulted in its application to studying fundamental biological processes. However, SMLM is a young and rapidly evolving field with multiple approaches to data acquisition and data analysis. In the context of SMLM, DBSCAN and Voronoï tessellation are extremely robust, widely used and highly optimized algorithms that allow the determination of nanomolecular clustering using fundamentally different approaches to arrive at similar conclusions regarding the geometry of molecular interaction. However, these algorithms have not been implemented to utilize the temporal information inherent to single-particle tracking SMLM. R-tree spatial indexing is a similarly robust and optimized algorithm that we have employed here to establish molecular overlap as a measure of potential clustering. The fundamental advantage of the NASTIC approach detailed in this study is that temporal information is used to inform the algorithm such that spatiotemporal clusters are intrinsic to the analysis rather than having to be derived subsequent to purely spatial clustering using approaches such as tcPALM (time-correlated photoactivated localization microscopy^9,52^). The NASTIC pipeline delivers a useful temporal dimension to SMLM analysis across a range of data geometries without dramatic increases in analysis time. The use of a trajectory-based approach allows MSD and VAR to further refine the analysis, and the ability to resolve spatiotemporal clusters opens a window into the dynamics of molecules within defined subcellular regions. The frameworks based on the abovementioned algorithms have their strengths and drawbacks, and all are reliant upon user metric guidance or empirical determination of optimal parameters. A major challenge when using SMLM clustering algorithms is to determine the parameters that will accurately capture the unknown ground truth of SMLM datasets. As for all commonly used analysis techniques, NASTIC requires optimized parameters suited to the geometry and density of the input trajectory data. This optimization necessitated the use of synthetic data generated using a wide range of detection densities, trajectory sizes, lengths, and data acquisition rates typical of current SMLM datasets. Due to the complex relationship between cluster metrics, we derived an optimization strategy based on determining which *r/t* pairs most closely returned the ground truth and further verified this strategy using additional statistical approaches, including Adjusted Rand Index (ARI) and Intersection over Union (IoU)^25^. NASTIC output robustly returned the ground truth over the large range of synthetic datasets tested and compared well with DBSCAN^14^ and Voronoï tessellation^15^ in our hands. As expected, in the case of extremely low or high data density, NASTIC, like other algorithms, progressively failed to return accurate clustering (Fig. 2h–l and Supplementary Fig. 10). Regardless of the clustering algorithm used, acquisition of high-quality single-molecule datasets are critical.

NASTIC has been used here to capture spatiotemporal clustering information from experimentally derived sptPALM and uPAINT data. We investigated the spatiotemporal dynamics of the SNARE protein syntaxin1 (Sx1a) in neurosecretory cells by sptPALM. Syntaxin plays a key role in mediating vesicular fusion in neurosecretory cells and neurons. With biological functions related to priming and fusion of secretory vesicles, which occur in the range of seconds to milliseconds, Syntaxin spatial clustering has been an area of intense scrutiny^31,50,51^. However, the temporal aspects of such clustering, likely to define syntaxin function in exocytosis, could not be defined in these previous studies. Using NASTIC, we were able to corroborate and expand upon the previously demonstrated Sx1a nanoscale organization. We found that Sx1a can form transient nanoclusters on discrete areas of the plasma membrane with an average lifetime of 12 s. Some of these clusters occurred multiple times in the same area of the membrane suggesting repetitive clustering behavior (hotspots). This advocates for scrutiny of the molecular underpinnings of clustering duration and hotspotting as another layer of regulation of biological processes through the precise temporal control of protein clustering. The regulation of Sx1a cluster formation and dissociation, and its ability to interact with other SNARE proteins and regulators in these discrete areas, remains enigmatic. Previous investigations into the temporal nature of nanoclusters have shown that they are indeed key to precisely control ligand binding and signaling duration^9,53^. The ability to accurately measure temporal clustering in a facile and unbiased way, therefore, opens up possibilities for exploring the role and regulation of these transient and potentially iterative clusters, along with their protein and lipid composition. Our current temporal analyses of hotspotting rely on statistically exploring the homogeneity of the detection density. Future work will be needed to extend these analyses to accurately extract additional hotspotting metrics.

A number of recent studies have applied NASTIC to investigate protein clustering dynamics in a range of biological systems. These include the co-receptor nanoclustering that mediates botulinum neurotoxin selective internalization into synaptic vesicles,^54^ the spatiotemporal dynamics of Fyn kinase in live hippocampal neurons,^55^ and identification and characterization of Synaptotagmin and SV2a co-clustering^56^.

In addition to its current applications, NASTIC has a number of potential future uses which are currently being explored. We have demonstrated in this study that spatiotemporal indexing can be expanded to multiple color SMLM analysis of different target proteins in the same cell^49^, where its ability to resolve hotspots may offer insights into cluster dependency, whereby one molecule may control the clustering of another. Two-color SMLM is an emerging technology with technical challenges; however, NASTIC2C is well suited to the analysis of these datasets. Finally, a particular advantage of the R-tree algorithm is that it is capable of functioning with an unlimited number of data dimensions. As such, the R-tree-based NASTIC could be leveraged to allow the analysis of 3D SMLM data consisting of three spatial dimensions and one temporal dimension, with fluorescence intensity as an additional dimension, for example.

SMLM is an inherently stochastic technology that, by necessity, only retrieves information on a small proportion of the molecular population. Thus, post-acquisition analysis techniques can critically only report an approximation of the dynamic molecular landscape. Techniques that can derive additional information from these limited SMLM datasets are therefore of great benefit. By robustly leveraging the temporal aspect, NASTIC offers additional much-needed insights into spatiotemporal clustering.

## Methods

### PC12 cell culture, transfection and plating

Pheochromocytoma (PC12) cells were maintained in Dulbecco’s Modified Eagle Medium (DMEM, containing sodium pyruvate) (Thermo-Fisher Scientific), fetal bovine serum (7.5%, Gibco) and horse serum (7.5%, Gibco), and 0.5% GlutaMax (Thermo-Fisher Scientific) at 37 ^o^C and 5% CO_2_. Cells were transfected by Lipofectamine®LTX with Plus Reagent (Thermo-Fisher Scientific) as per the manufacturers’ instructions with 2 µg of DNA or 1 µg of each plasmid when co-transfected. Cells were replated onto 0.1 mg/ml Poly-D-lysine (Sigma) on 29 mm No. 1.5 glass-bottomed petri dishes (Cellvis) 24 h post-transfection and imaged 48 h post-transfection. Live PC12 cells were imaged in the isotonic condition in buffer A (145 mM NaCl, 5 mM KCl, 1.2 mM Na_2_HPO_4_, 10 mM D-glucose and 20 mM Hepes, pH 7.4) at 37 °C.

### Plasmids and fluorescent nanobodies

pmEos2-Munc18-1 (SNM) (Munc18-1-mEos2), pmEos2-N1 syntaxin 1a (Sx1a-mEos2), and pEGFP-N1 syntaxin 1a (Sx1a-EGFP) have been previously described^11^. Fluorescently labeled antibodies (Synaptic Systems, anti-GFP Atto647N tagged nanobodies, Cat#: GFP sdAb - FluoTag-Q - N0301-At647N-L) were reconstituted as per the manufacturer’s instructions and utilized at 3.19 pg/μl in live uPAINT experiments.

### SMLM acquisition

PC12 cells transfected with Sx1a-mEos2 or Munc18-1-mEos2 were analyzed by sptPALM. PC12 cells transfected with Sx1a-EGFP were analyzed by universal point accumulation in nanoscale topography (uPAINT) as described in ref. ^48^, and tracked using anti-GFP Atto647N tagged nanobodies^46,57,58^ at 3.19 pg/µl.

Live transfected cells were visualized on a Roper Scientific Total Internal Reflection Fluorescence (TIRF) microscope equipped with an iLas 2 double-laser illuminator (Roper Scientific), a Nikon CFI Apo TIRF ×100/1.49 NA oil-immersion objective, and an Evolve 512 Delta EMCCD camera (Photometrics). Time-lapse TIRF movies (16,000 frames) were captured at 50 Hz for ~320 s at 37 °C.

For single-particle tracking photoactivated localization microscopy (sptPALM) analysis, samples were illuminated simultaneously with a 405 nm laser (Stradus, Vortan Laser Technology) to photoactivate mEos2-tagged proteins, and a 561 nm laser (Jive, Cobolt Lasers) for excitation of the photoconverted mEos2. A double-beam splitter (LF488/561-A-000, Semrock) and a double-band emitter (FF01-523/610-25, Semrock) were used to isolate the mEos2 signal from autofluorescence and background signals. To achieve optimal spatial and temporal separation of stochastic mEos2 blinking, the power of the 405 nm and 561 nm laser was adjusted to 7.13 mW/cm^2^ and 2.04 × 10^5^ mW/cm^2,^ respectively, at the focal plane.

For uPAINT, experiments were performed according to Giannone^48^. An anti-GFP nanobody^57^ tagged with ATTO 647N-NHS-ester (Atto-Tec GmbH) was used to track Sx1a-EGFP single molecules. PC12 cells were double transfected with Munc18-1-mEos2 and Sx1a-EGFP to perform dual-color imaging. ATTO 647N–tagged anti-GFP nanobodies were added at 1 nM for low-level stochastic labeling. Time-lapse TIRF movies (16,000 frames) were recorded at 50 Hz at 37 °C on a TIRF microscope (Roper Technologies) equipped with an ILas2 double-laser illuminator (Roper Technologies). Each cell was imaged in both control condition and stimulated condition (2 mM BaCl_2_). A quadruple beam splitter (LF 405/488/561/635-A-000-ZHE; Semrock) and a quad band emitter (FF01-446/510/581/703-25; Semrock) were used for illumination. The power of the 642 nm laser used was 60% of the initial laser power (2.16 × 10^5^ mW/cm^2^ measured at the focal plane).

All SMLM data were acquired using MetaMorph (Meta-Morph Microscopy Automation and Image Analysis Software, version 7.7.8; Molecular Devices) and further processed using the PalmTracer software^59^. NASTIC uses trajectory data in the TRXYT format, consisting of four header-less space-separated columns corresponding to trajectory number, *x* coordinate (µm), *y* coordinate (µm) and detection time (s). In this study, PalmTracer output was converted to TRXYT format using a custom Matlab script. Typical TRXYT data:

1 9.0117 39.86 0.02

1 8.9603 39.837 0.04

1 9.093 39.958 0.06

1 9.0645 39.975 0.08

etc.

### Software

While initial investigations were performed using multiple command line driven Python scripts, we subsequently consolidated the analysis into a suite of single-script graphic user interfaces (GUIs) suitable for general use by non-programmers: *NASTIC* (nanoscale spatiotemporal indexing clustering), *Segment NASTIC (segNASTIC)*, *Two-color NASTIC (NASTIC2C)* and *two-color segNASTIC (segNASTIC2C)*. The programs, collectively referred to as *NASTIC*, require Python 3.8 or greater, and a number of modules which can be installed using:

python -m pip install scipy numpy matplotlib matplotlib-venn pysimplegui rtree scikit-learn statsmodels colorama

The GUI was constructed using the *tk* version of *PySimpleGui* (https://pysimplegui.readthedocs.io/). *NASTIC* was developed under and should run without issues on 64 bit Windows 10 and Windows 11. It will run with minor *tk* interface anomalies under Linux, but the authors cannot guarantee optimum performance under MacOS. NASTIC uses the Python *multiprocessing* module to fork computationally intensive operations onto all available cores of the physical or virtual machine on which it runs. This results in a significant increase in performance but precludes packaging into standalone executables using *PyInstaller*, and *NASTIC* will therefore only run on a computer with a Python 3.8+ environment.

The *NASTIC* interface divides the analysis workflow into a series of tabs that broadly allow the user to: (1) load, screen and display the detections from an SMLM TRXYT file; (2) select or load one or more rectangular or irregular regions of interest (ROIs) and optionally adjust the density of selected trajectories within the ROIs; (3) adjust clustering parameters and apply NASTIC to the selected trajectories; (4) display and save the results of the clustering with a high degree of control over the presentation of trajectories, centroids and clusters; and (5) run a series of post clustering analyses to visualize metrics such as segment overlap, MSD, diffusion coefficient and cluster overlap probabilities, and save tabulated data for downstream comparative analyses. Typical visualizations are shown in Figs. 3 and 4. Visualized data are saved as 300 dots per inch (dpi) PNG files, and the main clustering images can be additionally saved at a user-specified dpi in a range of user-specified file formats (EPS, PDF, PNG, PS, SVG, TIF). The coordinates of the ROIs are saved as tab separated *roi_coordinates.tsv*: ROI, *x* coordinate (µm), *y* coordinate (µm), which can be loaded back into the *NASTIC* ROI tab to facilitate future analyses. Analysis metrics are saved as tab separated *metrics.tsv*, which can be viewed in any spreadsheet or text editor, and further processed for comparative studies using *NASTIC Wrangler* or other software.

Comparative analysis of multiple tabulated data files (generated during step 5 above) from two experimental conditions was performed using another simple Python GUI designated *NASTIC Wrangler*. This program allows the user to specify two directories, each consisting of further subdirectories containing saved tabulated metrics data. *NASTIC Wrangler* recursively reads and compiles the tabulated metrics data from the subdirectories of each specified directory and outputs a series of annotated comparative bar plots, thereby allowing the user to examine the degree and significance of the change of a number of spatiotemporal clustering metrics. The significance of any differences in bar plots between conditions is determined by unpaired two-tailed Student’s *t*-test.

### Statistics & reproducibility

Statistical analyses of clustering metrics returned by NASTIC were performed via *t-*test as described above. Kolmogorov–Smirnov (KS) was used to test for uniformity of distribution of trajectory centroids with respect to *x*- and *y*- axes. Statistical tests are indicated in the figure legends. Data were considered significant at *p* < 0.05 and are displayed such that ns = no significance, *p < 0.05, **p < 0.01, ***p < 0.001, ****p < 0.0001.

## Supplementary information

Editorial Assessment Report

Supplementary Figures

## Source data

Source data

## Data Availability

The TRXYT data used during this study is available for download from the publicly accessible institutional data repository of The University of Queensland (UQ eSpace) 10.48610/0901bca. Source data are provided with this paper.

## Code availability

All Python codes used in this study are available at the GitHub repository https://github.com/tristanwallis/smlm_clustering. The v1.0.0 release of the *NASTIC* suite is available at 10.5281/zenodo.7847776. *NASTIC* and associated scripts are released under a Creative Commons CC BY 4.0 licence: you are free to use and modify the code on the proviso that you make any changes freely available, acknowledge the original authors in derivative works and do not release said works under a more restrictive licence.
