# Author Correction: Super-resolved trajectory-derived nanoclustering analysis using spatiotemporal indexing

**DOI:** 10.1038/s41467-023-40261-6

**Published:** 2023-07-25

**Authors:** Tristan P. Wallis, Anmin Jiang, Kyle Young, Huiyi Hou, Kye Kudo, Alex J. McCann, Nela Durisic, Merja Joensuu, Dietmar Oelz, Hien Nguyen, Rachel S. Gormal, Frédéric A. Meunier

**Affiliations:** 1 Clem Jones Centre for Ageing Dementia Research, Queensland Brain Institute, The University of Queensland, Brisbane, QLD 4072 Australia; 2 School of Mathematics and Physics, The University of Queensland, Brisbane, QLD 4072 Australia; 3 Queensland Brain Institute, The University of Queensland, Brisbane, QLD 4072 Australia; 4 School of Biomedical Sciences, The University of Queensland, Brisbane, QLD 4072 Australia; 5 Present Address: Australian Institute for Bioengineering and Nanotechnology, The University of Queensland, Brisbane, QLD 4072 Australia

**Keywords:** Image processing, Functional clustering, Super-resolution microscopy

Correction to: *Nature Communications* 10.1038/s41467-023-38866-y, published online 08 June 2023

In this article the grant number R21AG080435 relating to the National Institute on Aging of the National Institutes of Health for Frédéric A. Meunier was omitted. The original article has been corrected.

