# Supplementary material for: Super-resolved trajectory-derived nanoclustering analysis using spatiotemporal indexing
Source: Nat Commun. 2023 Jun 8;14:3353. doi: 10.1038/s41467-023-38866-y (PMC10250379; doi:10.1038/s41467-023-38866-y)
Supplement: Supplementary file 1 — Editorial Assessment Report [file 41467_2023_38866_MOESM1_ESM.pdf]

Contents of this report

1. **Manuscript details**: overview of your manuscript and the editorial team.
2. **Review synthesis**: summary of the reviewer reports provided by the editors.
3. **Editorial evaluations**: personalized evaluation and recommendation from all 3 journals.
4. **Annotated reviewer comments**: the referee reports with comments from the editors.
5. **Open research evaluation**: advice for adhering to best reproducibility practices.

About the editorial process

Because you selected the **Nature Portfolio Guided Open Access** option, your manuscript was assessed for suitability in three of our titles publishing high-quality work across the spectrum of methods research: ***Nature Methods***, ***Nature Communications***, and ***Communications Biology***. More information about Guided Open Access can be found here.

Collaborative editorial assessment

Your editorial team discussed the manuscript to determine its suitability for the Nature Portfolio Guided OA pilot. Our assessment of your manuscript takes into account several factors, including whether the work meets the **technical standard** of the Nature Portfolio and whether the findings are of **immediate significance** to the readership of at least one of the participating journals in the Nature Portfolio Guided Open Access methods cluster.

Peer review

Experts were asked to evaluate the following aspects of your manuscript:

- **Novelty** in comparison to prior publications;
- **Likely audience** of researchers in terms of broad fields of study and size;
- **Potential impact** of the study on the immediate or wider research field;
- **Evidence** for the claims and whether additional experiments or analyses could feasibly strengthen the evidence;
- **Methodological detail** and whether the manuscript is reproducible as written;
- Appropriateness of the **literature review**.

Editorial evaluation of reviews

Your editorial team discussed the potential suitability of your manuscript for each of the participating journals. They then discussed the revisions necessary in order for the work to be published, keeping each journal's specific editorial criteria in mind.

Journals in the Nature portfolio will support authors wishing to transfer their reviews and (where reviewers agree) the reviewers' identities to journals outside of Springer Nature.
If you have any questions about review portability, please contact our editorial office at guidedoa@nature.com.

nature portfolio

GUIDED OPEN ACCESS

nature methods | nature communications | communications biology

Manuscript details

Tracking number	Submission date	Decision date	Peer review type
GUIDEDOA-21-00359	Dec 10, 2021	Feb 22, 2022	Single-blind

Manuscript title

Molecular videogaming: super-resolved trajectory-derived nanoclustering analysis using spatio-temporal indexing

Preprint: link if available

Author details

Frederic Meunier

Affiliation: University of Queensland

Editorial assessment team

**Primary editor**	**Rita Strack**<br>Home journal: ***Nature Methods***<br>ORCID: **0000-0003-1845-7116**<br>Email: **rita.strack@us.nature.com**
**Other editors consulted**	**Cara Eldridge**<br>Home journal: ***Nature Communications***<br>ORCID: **0000-0001-7001-2312**<br><br>**Anam Akhtar**<br>Home journal: ***Communications Biology***<br>ORCID: **0000-0002-8820-8468**
**About your primary editor**	Rita Strack obtained her Ph.D. in Biochemistry and Molecular Biology from the University of Chicago. While there, she worked with Benjamin Glick and Robert Keenan to engineer improved variants of the red fluorescent protein DsRed, and also studied the chemical mechanism of chromophore formation in DsRed. She continued her research as a postdoctoral fellow in Samie Jaffrey's laboratory at Weill Cornell Medical

	College, where she developed fluorescent reporters for live-cell imaging of RNA such as Spinach2. She handles imaging, microscopy and probes, along with protein and RNA biochemistry content for the journal. Rita joined Nature Methods in November 2014.

Editorial assessment and review synthesis

Editor's summary and assessment	This paper describes a new clustering algorithm for single-molecule localization microscopy data that is inspired by algorithms used in video games for determining whether objects are clustered together. The authors note that existing methods like DBSCAN and Voronoi tessellation do not work well to examine clustering data that has a temporal dimension, like looking for changes in clustering over time during live imaging.<br><br>Thus they created the molecular clustering pipeline, NAnoscale SpatioTemporal Indexing Clustering (NASTIC), which efficiently returns spatiotemporal clusters and associated metrics from time-lapse SMLM data.<br><br>The authors demonstrate NASTIC on synthetic data and compare its output to DBSCAN and Voronoï tessellation with favorable results. They also demonstrated on sptPALM data of Syntaxin 1a-mEos2 in neurons and were able to resolve hotspots of nanocluster formation. They further show the approach works for high density SMLM data and for two-color experiments.<br><br>The editors thought the conceptual and technical advance was sufficient to justify sending the paper for peer review as part of the GOA trial.

**Editorial synthesis of reviewer reports**	The reviewers found the method of interest, but had many technical concerns and concerns about broad applicability of the approach. The reviewers raised concerns about the performance of the method in terms of parameterization, and about performance relative to other tools meant for handling clustering data with a temporal dimension. The referees also wanted additional experiments to strengthen the claims made by the authors.

Editorial recommendation

Nature Methods<br><br>Revision not invited	While the referees had some positive things to say about the work, the editors at Nature Methods do not feel it represents enough of an advance over existing tools in terms of enabling new biological discovery to justify further consideration.
Nature Communications<br><br>Major revisions with extension of the work	We agree with the referees that there are a number of major concerns that must be addressed for us to continue the review process at Nature Communications. Our concerns include the performance of your method in terms of parameterisation, the performance relative to other tools, and the use of simulated data. We would require all of these comments to be addressed, and the superiority of your method over existing methods to be shown. For further information please see our comments below.
Communications Biology<br><br>Major revisions	While we are happy to forgo some requests, like some of the parameterisations concerns by the reviewers and the application on actual data, we do ask for further benchmarking and addressing other comments as completely as possible. Hence, we invite for major revisions.

Next steps

**Editorial recommendation 1:**	Our top recommendation is to revise and resubmit your manuscript to *Nature Communications.* We do require an extension of the manuscript including new experiments and this will require a significant amount of work.
**Editorial recommendation 2:**	You may also choose to revise and resubmit your manuscript to *Communications Biology.* This option might be best if the requested experimental revisions are not possible/feasible at this time.
**Note**	As stated on the previous page *Nature Methods* is not inviting a revision at this time. Please keep in mind that the journal will not be able to consider any appeals of their decision through Guided Open Access.

Revision

To follow our recommendation, please upload the revised manuscript files using **the link provided in the decision letter**. Should you need assistance with our manuscript tracking system, please contact Adam Lipkin, our Nature Portfolio Guided OA support specialist, at guidedOA@nature.com.

Revision checklist

- ☐ Cover letter, stating to which journal you are submitting
- ☐ Revised manuscript
- ☐ Point-by-point response to reviews
- ☐ Updated Reporting Summary and Editorial Policy Checklist
- ☐ Supplementary materials (if applicable)

Submission elsewhere

If you choose not to follow our recommendations, you can still take the reviewer reports with you.

Option 1: Transfer to another Nature Portfolio journal
Springer Nature provides authors with the ability to transfer a manuscript within the Nature Portfolio, without the author having to upload the manuscript data again. To use this service, **please follow the transfer link provided in the decision letter.** If no link was provided, please contact guidedOA@nature.com.

> *Note that any decision to opt in to In Review at the original journal is not sent to the receiving journal on transfer. You can opt in to In Review at receiving journals that support this service by choosing to modify your manuscript on transfer.*

Option 2: Portable Peer Review option for submission to a journal outside of Nature Portfolio
If you choose to submit your revised manuscript to a journal at another publisher, we can share the reviews with another journal outside of the Nature Portfolio if requested. You will need to request that the receiving journal office contacts us at guidedOA@nature.com. We have included editorial guidance below in the reviewer reports and open research evaluation to aid in revising the manuscript for publication elsewhere.

Annotated reviewer reports

The editors have included some additional comments on specific points raised by the reviewers below, to clarify requirements for publication in the recommended journal(s). However, please note that all points should be addressed in a revision, even if an editor has not specifically commented on them.

Reviewer #1 information

**Expertise**	Single-molecule imaging, super-resolution imaging, quantitative analysis of SMLM data
**Editor's comments**	The referee found the method potentially useful, but wanted to see major revisions prior to publication. These include additional experiments, specifically 2-color SMLM experiments.

Reviewer #1 comments

**Section**	**Annotated Reviewer Comments**
**Remarks to the Author: Overall significance**	The authors have developed a method based on spatial indexing that incorporates the time dimension (NASTIC) to analyze the spatial and temporal overlap of single molecule trajectories obtained from SMLM experiments. By comparing with current algorithms used in SMLM clustering analysis (DBSCAN and Voronoi tessellation), the authors establish the additional information that becomes now accessible with NASTIC as well as its superior performance to the state of the art. The authors use NASTIC to perform spatiotemporal clustering analysis of syntaxin 1a and Munc18-1 and identify hotspots that could play a role in biological function. Furthermore, the analysis of trajectory segments (segNASTIC) and of two color experiments for heterocomplexes, provide useful extension that increase the quality of the analysis achievable with this approach.
**Remarks to the Author: Strength of the claims**	A number of concerns should be addressed to improve the quality of the work:<br>- Could the authors estimate to which extent the idealized bounding box accurately represents the trajectory data? what is the error of this approximation?<br>**Both *Nature Communications* and *Communications Biology* would require you to address these concerns.**<br>- How does the definition of cluster in terms of % of spatial and temporal overlap affect the analysis of the data?<br>**Understanding how this definition may affect the data analysis is required for both *Nature Communications* and *Communications Biology*.**<br>- How do the optimal range of r and t values change with the length of the trajectory and the temporal resolution, as well as the diffusion coefficient of the particles? And how does their goodness change with these parameters?<br>**Please address these concerns for both *Nature Communications* and *Communications Biology*.**<br>- How well does the approach work with heterogeneous data (in terms of mobility and length of the trajectories)?<br>**Please show how your approach works for *Nature***

Communications. You can address this textually for _Communications Biology_.

- Concerning the Convex hull of the trajectories: It is not clear why the radius factor r needs to be larger than 1 to create a "bounding box more representative of the full extent of the trajectory". I was wondering if a relative bounding box of each step of a trajectory would not be more exact. Then it is based on the MSD between two localizations in two consecutive frames. This would include the uncertainty of unknown localization of the protein of interest between two localization points of a trajectory. Finally, this will impact the convex hull, and can be an improvement for a better hotspot identification. In addition, implementing a bounding box of each trajectory step, will possibly take the uncertainty of localization of the protein of interest between two consecutive frames (two localizations identified) into account and result in a more precise convex hull of the trajectory. In contrast to this, a relative convex hull of the trajectories would also be an interesting aspect. Did the authors think about this? And if so, why did they decide for a non-relative convex hull?

Please fully address all of these parameterisation concerns for _Nature Communications_. Please address this textually by discussing it for _Communications Biology_.

-

These and other issues should also be considered in the comments to the figures:

- To Fig. 2 and Fig. S1:
Would a r≤ 1.2 and different t (t=1 s, t=5 s, t=10 s, t=20 s) also allow detecting sub-clusters? In Fig. 2aiii (green circle) it seems that there are sub-clusters. If sub-clusters are detected as one cluster, is the chance to detect a hotspot higher?

For both _Nature Communications_ and _Communications Biology_, we ask that you show how sub-cluster detection would work with your method.

- Trajectories which are part of in different clusters should be identified. Thus, a confinement time can be measured - so how long does a S1a-mEO2 signal/protein stay inside a cluster? Confined molecules get bleached faster, here using less laser power (<22 mW) would be interesting. Otherwise the authors can provide data to show why the used laser powers of 405 nm (4mW) and 561 nm (22 mW) are that optimal for the spatial and temporal separation.

For _Nature Communications_ we ask that you address the comment of using a lower laser power. For _Communications Biology_, kindly explain why the laser powers used were optimal for the purpose.

- Furthermore, can also trajectories be identified which do not get confined in a cluster but perform a "kiss and run" behavior?

For _Nature Communications_ we would require additional data to assess the 'kiss and run' behaviour. Please address this textually for _Communications Biology_.

- Concerning the sptPALM measurements, the cluster identification depends on the confined trajectories. Here a higher frame rate would be interesting, in order to test for an even more accurate tracking of the signals in order to have a higher precision in the cluster analysis.

For _Nature Communications_ we ask that you use a higher frame rate as suggested here. It is not mandatory to address this concern for consideration at _Communications Biology_.

- Regarding the two-color SMLM analysis, it is only performed with simulated data. Here it would be of great benefit to perform/use a real two-color

	experiment/dataset of two proteins which either directly or indirectly interact with each other, to reveal the co-clustering of these proteins in one cluster in a time dependent manner.<br>***Nature Communications* would require you to perform the two-colour SMLM analysis on real data, as suggested by the reviewer. At *Communications Biology*, we are okay to forego this request.**
**Remarks to the Author: Reproducibility**	***Nature Communications* and *Communications Biology* require you to address all concerns regarding reproducibility.**<br>To Fig, S1:<br>It would be good to see what results are acquired with r 1.2 and t= 10 s . It's not possible to see the centroid of a trajectory represented as a dark dot, please use a color (like bright orange) to show this. The trajectories are also not visible. The use of a white background would solve this. It is not possible to understand where a trajectory starts and ends, this would be necessary to understand the outcome of the figure and thus for the r value and time value finally used.<br><br>Fig. 3e, the color bar of the diffusion coefficients should be bigger and include more numbers, especially -3, as most of the diffusion coefficients (Fig. 3g) seem to appear in this range.<br><br>Fig. 3f, an additional view of the data-set like in Fig. 4e-f would be a benefit to the figure in order to show the overlap of confined trajectories, as a final version this may also include an animation in 3D including a sliding window

Reviewer #2 information

**Expertise**	Quantitative analysis of SMLM data, including cluster analysis
**Editor's comments**	The referee thought the work was potentially of interest to *Nature Methods*, but raised important concerns about parameter choices and benchmarking.

Reviewer #2 comments

Section	Annotated Reviewer Comments
**Remarks to the Author: Overall significance**	This manuscript presents NASTIC, a collective software for SMLM data analysis. NASTIC leverages spatial and temporal information for molecular clustering in living cells. The overlap of the bounding boxes of molecule trajectories is determined by the R-tree spatial indexing algorithm. Additionally, the temporal information of the bounding box is extended via a user-defined parameter, thickness, for the tracked molecule duration. The authors validated NASTIC on simulated data that is generated for the sake of parameters tuning. After that, NASTIC has been used for molecular clustering in living cells imaged using various SMLM imaging methods.<br><br>The manuscript lacks the state-of-the-art methods (literature review) usually

	used in cluster analysis for super-resolution microscopy. There are several works that leveraged temporal information for molecular clustering in both living and fixed cells. For example, see the below non-exhaustive list of papers:<br>https://dx.doi.org/10.1002/smtd.201800008<br>https://dx.doi.org/10.1038/nmeth.1704<br>https://dx.doi.org/10.1038/nmeth.3897<br>https://dx.doi.org/10.1038/nmeth.4065<br>https://doi.org/10.1039/C9NR08594G<br>https://dx.doi.org/10.1038/s43586-021-00038-x<br>http://dx.doi.org/10.1002/9781119096948.ch8<br>**For *Nature Communications* we ask that you include this additional literature in your manuscript, and add thorough benchmarking of your work. For *Communications Biology*, we ask that you cite these approaches in the paper and benchmark your method against at least one of the most relevant approaches (see similar point from Reviewer 3).**
**Remarks to the Author: Impact**	The manuscript is appropriate for the nature methods journal, as it proposes a new clustering method for single molecule localization microscopy (SMLM) data. However, the manuscript requires addressing major comments before proceeding to publication. Some comments require designing new experiments and simulation scenarios. Additionally, the current comparison with the state-of-the-art methods is not complete and biased toward NASTIC.
**Remarks to the Author: Strength of the claims**	Major points:<br><br>1) On page 5 - 3rd paragraph. NASTIC parameter selection is somehow subjective. I think this is not a good way to show how the parameters are selected given the ground truth (GT) data. The authors should show a graph/matrix/heatmap for a range of r and t values with respect to an evaluation measure such as adjusted mutual information (AMI) or adjusted rand index (ARI). The pair of parameters are then selected based on a measure that achieves the best performance/clustering.<br>**For both *Nature Communications* and *Communications Biology*, we require you to address the parameterisation concerns raised by this reviewer.**<br><br>2) On page 5 - 4th paragraph and Supp. Fig. 2. The authors do not justify the way they select the parameters from the r/t graph. Why is the inflection point important? "We looked for the inflection point where these lines transitioned from vertical to horizontal.". Also, it seems the way the parameters are selected is not accurate. The 'pale' color can be found in many pairs of (r,t). For example, there are rows of radii for the same t that are pale and the same thing for columns of t for the same r have a pale color. I find it hard to find r and t from this figure. I suggest using other measures such as AMI and ARI to find the optimal parameters for clustering.<br>**For both *Nature Communications* and *Communications Biology*, we require you to address the parameterisation concerns raised by this reviewer.**<br><br>3) On page 5 - 4th paragraph. "In all subsequent NASTIC analyses, r = 1.2 and t = 20 s were used as the default parameters.". Again, these parameters are based on simulated data. The authors should do the simulation with different scenarios (e.g., various number of trajectories, various cluster sizes,

	various densities, various noise levels, etc.) and then see if these parameters do not change much along with all the scenarios. Then, the default parameters can be defined and justified. Moreover, the authors keep using the default parameters when moving to live cells analysis. I think the parameters need to be justified based on biological knowledge besides the heuristics derived from the simulated data.<br>**_Nature Communications_ requires you to optimise the parameters based on biological information, rather than using the simulated data throughout. For _Communications Biology_, you can textually address this concern and explain why according to you, the parameters selected are the most appropriate.**<br><br>4) On page 6 - 1st, 2nd paragraphs, and Fig 2. For a fair comparison, the parameters of all methods should be selected using a search method based on the GT data. Otherwise, it is not a fair comparison. To show the added value of the new clustering method, it should be compared fairly with the state-of-the-art methods leveraged for SMLM data analysis. You might get an idea on how to select the optimal parameters, for example, for the DBSCAN method for SMLM data analysis from the below paper:<br>https://doi.org/10.1101/2021.06.19.449098<br>**Both _Nature Communications_ and _Communications Biology_ require comparison with existing methods, as suggested here.**<br><br>5) In Fig. 2d-g, the cluster features are coupled with the clustering method. If the method parameters are not optimally selected, then the resulting cluster features will not reflect the actual cluster features. I suggest selecting DBSCAN and Voronoi clustering methods parameters using the ARI or AMI measures. Then, find the cluster features and compare them with NASTIC cluster features. Consequently, I find the below result not complete until the authors tune the parameters of the other clustering methods as described in the previous comments. (from the last paragraph of page 6) "NASTIC consistently returned metrics most closely matching the ground truth of the simulated data."<br>**We ask that you address this concern for both _Nature Communications_ and _Communications Biology_.**<br><br>6) On page 7 - 2nd paragraph and Supp. Fig 3. How are the parameters selected? Are they based on simulated data? I think you can validate the algorithm based on simulated data. However, for live cells, the parameters should be selected based on some biological/photophysical knowledge or observations as well.<br>**As above, we ask that you address these concerns on the parameters for _Nature Communications_. We are okay to forego this request for _Communications Biology_.**<br><br>7) On page 9 - 2nd paragraph. "Across all segments in an acquisition, a histogram was generated showing that the majority of trajectory segments had low overlap (Supp. Fig. 6)." For low-density clusters (with few localizations/trajectories), the trajectories overlapping might be very low. How about various cluster densities data? Have you tested the robustness of NASTIC to cluster this kind of data?<br>**_Nature Communications_ ask that you extend your manuscript to address how your method performs with this type of data. We are fine to forego this reviewer request for _Communications Biology_.**

	8) Comparing the spatiotemporal clustering method (i.e., NASTIC) with the spatial clustering methods (e.g., DBSCAN) is not fair. It should be compared with other temporal clustering methods and moreover, should be compared with other spatiotemporal methods. It is worth doing a comparison with the SMLM clustering method designed for living cells beside the DBSCAN and Vornois based methods covered in the manuscript. For example, NASTIC should be compared with some related works from the following papers: https://dx.doi.org/10.1002/smtd.201800008; https://dx.doi.org/10.1038/s41592-021-01154-y; https://doi.org/10.1101/2021.06.19.449098<br>**As before, we require additional benchmarking for *Nature Communications* and *Communications Biology*.**<br><br>Minor points:<br><br>1) The term 'nanocluster' should be revisited and defined clearly in the manuscript. The 'nanocluster' term was already defined in many published papers. It is related to the problem of cluster artifacts generated by overcounting of blinking fluorophores (e.g., https://dx.doi.org/10.1038/nmeth.3897; https://dx.doi.org/10.1038/nmeth.4065). However, in this manuscript, the term is not consistent with the definition used in the literature.<br>**Both *Nature Communications* and *Communications Biology* require you to define your use of the term 'nanocluster'.**<br><br>2) In the Results section, page 4, I suggest adding a pseudo-code or pipeline that could show the stages of the NASTIC algorithm. It would be much better than the textual description.<br>**We agree that this is a good suggestion.**<br><br>3) n Fig. 2 and Supp. Fig. 1, the ground truth (GT) data should be provided/visualized to assess the quality of the clustering with respect to GT.<br>**Please add the ground-truth data for both the journals.**<br><br>4) In all the presented experiments (simulation and live cell), the authors captured movies at 50 Hz for 320 s (i.e., 16,000 frames). Can the authors apply NASTIC to analyze data with 30,000 or 50,000 or so frames with a relatively large field of view? This is to show the robustness of NASTIC to analyze big data. Also, this can be used to show the performance of NASTIC in terms of speed compared with other clustering methods. Moreover, this is very important for testing the sensitivity of NASTIC parameters to analyze the acquired data.<br>**As suggested by this reviewer, *Nature Communications*, ask that you test your method with a relatively large FOV, and show speed comparison to existing methods. Please discuss this point textually for *Communications Biology*.**
	The authors applied statistical tests when needed in the manuscript/figures

Reviewer #3 information

**Expertise**	Quantitative analysis of SMLM data, including cluster analysis
**Editor's comments**	The referee was largely convinced the approach works as described, but finds it simplistic and lacking in broad applicability. They also thought the work lacked appropriate benchmarking. The referee tested the software and found it in good working order.

Reviewer #3 comments

Section	Annotated Reviewer Comments
**Remarks to the Author: Overall significance**	In this work by Wallis et al., the authors present a new SMLM analysis method, called (seg)NASTIC, specifically designed to take into account the temporal information inherently provided by tracks. By defining a combination of temporal windowing and bounding box overlap, they manage to identify hotspots, i.e. areas where recurrent molecular clustering happens. While the authors are right that taking into account the temporal information provided by SMLM is important, and that most spatial clustering methods don't, I think that the technique presented here is too limited and too simple to be of wide interest for the field. Indeed, NASTIC only allows to identify hotspots, a specific and limited application, and the way the technique is designed makes it difficult to extend to extract other relevant information.<br><br>Comparison<br><br>The authors choose to compare their method to two well-known spatial clustering technique, which were not designed to take into account the temporal information. As such, NASTIC always outperforms them since the aim is to identify hotspots. Even if not many, there exist techniques that account for the temporal information of tracks. For instance, the group of Jean-Baptiste Masson have developed several tools with this objectives in mind (InferenceMAP, https://www.nature.com/articles/nmeth.3441 ; TRamWAy, https://research.pasteur.fr/fr/software/tramway-platform/). While I'm not familiar with those, I think that InferenceMAP can compute a range of different quantifications on tracks, from which determining hotspots is possible. How does NASTIC compare to InferenceMAP?<br>**As asked by the previous reviewers, *Nature Communications* and *Communications Biology*, would require this additional benchmarking for further consideration of your manuscript.**<br><br>Missing Reference<br>Beheiry, M., Dahan, M. & Masson, JB. InferenceMAP: mapping of single-molecule dynamics with Bayesian inference. Nat Methods 12, 594–595 (2015). https://doi.org/10.1038/nmeth.3441
**Remarks to the Author: Impact**	Unfortunately, I don't think that the proposed method will have a strong impact on the community, mostly because the technique is very simplistic and limited. Determining hotspots is, in the end, very easily performed as a

	post-processing step and I'm not convinced that doing it at the same time of the analysis is that essential.<br><br>Thus I think that Communications Biology is the most suited journal.
**Remarks to the Author: Strength of the claims**	While the method certainly works, I think that there are several shortcomings in its design, as well as a very limited scope.<br><br>1. Even if the technique is very simple, the definition of the bounding box is not well explained. I don't understand why the authors are using an idealized bounding box based on an approximated radius (if a radius is used, the bbox is not rectangular but square, isn't it?), and not the actual bbox. Depending on the track shape, this radius may or may not be not a good approximation of it. Also, except if I missed it, I don't think that the way this radius is computed is explained. Why not using the track bbox? If it's performance wise, I don't think that testing the bbox instead of a radius will have much effect on the algorithm run time. In addition, using a radius adds a parameter that could be discarded by using the real bbox.<br>**Both the journals require an explanation of your bounding box method and its performance.**<br><br>2. One feature of NASTIC highlighted in the manuscript is that identifying temporally distinct clusters is an intrinsic feature of the technique, and not a post-processing step. While I would agree if the analysis was more statistically relevant, the fact is that it is a simple temporal windowing. Thus, if you apply the same temporal windowing parameter to a spatial cluster, you will end up with the same temporal delimitation of the sub-clusters. And the authors still used DBSCAN to compute some clustering metrics. So why not applying the temporal window directly on the identified DBSCAN clusters?<br>**Please address this concern for both *Nature Communications* and *Communications Biology*.**<br><br>3. Figure 3h is used to validate that the clusters are more constrained in hotspots. But again, that also means that localizing hotspots can be easily retrieved by plotting the MSD, determining the constrained tracks and identifying where they are located. Unfortunately, it shows that the added value of NASTIC is limited, as this can be done in a few lines of Python code.<br>**Please comment on this concern for both the journals.**<br><br>4. I think that the effect of the t parameter was not accessed enough. The authors used some simulations with which they found that t=20 seconds was the best choice. While this value worked with their simulations/experimental data, I would expect that this parameters would be quite dependent on the average track duration, a parameter linked to the SMLM modality and biological model used.<br>**Please give further information on the parameterisation and the optimisation needed for applying this to non-simulated data for *Nature Communications*. Please address this textually for *Communications Biology*.**<br><br>5. I'm not sure what is the aim of having NASTIC and segNASTIC. The manuscript fails to differentiate the two, apart from the expected fact that segNASTIC will identify smaller clusters. I don't see the point of presenting a declination of the main technique if its interest is not illustrated in the manuscript.

	**Please give more information on the difference between NASTIC and segNASTIC and the importance of this for both the journals.**
**Remarks to the Author: Reproducibility**	The work presented in this manuscript is reproducible as the authors have released the source code of the method.<br><br>I was pleasantly surprised that NASTIC is properly packaged with GUIs, and that I was able to easily installed it in a conda environment. Everything seems to work as expected, and using Matplotlib allows to generate camera-ready Figures. Two minor comments: I would have preferred more interaction possibilities with the viewer. Every modification needs a button click to be generated/viewed, and I prefer to be able to zoom and move directly on the viewer. Interacting with the viewer created when defining multiple ROIs distant from each other can be a little weird as the viewer will use the bbox of all the ROIs, and because you cannot directly zoom.

Open research evaluation

Guidelines for Transparency and Openness Promotion (TOP) in Journal Policies and Practices ("TOP Guidelines")

The recommendations and requests in the table below are aimed at bringing your manuscript in line with common community standards as exemplified by the TOP Guidelines. While every publisher and journal will implement these guidelines differently, the recommendations below are all consistent with the policies at Nature Portfolio. In most cases, these will align with TOP Guidelines Level 2.

FAIR Principles

The goal of the recommendations in the table below related to **data or code** availability is to promote the FAIR Guiding Principles for scientific data management and stewardship (*Scientific Data* **3**: 160018, 2016). The FAIR Principles are a set of guidelines for improving 4 important aspects of digital research objects: **F**indability, **A**ccessibility, **I**nteroperability and **R**eusability.

ORCID

ORCID is a non-profit organization that provides researchers with a unique digital identifier. These identifiers can be used by editors, funding agencies, publishers, and institutions to reliably identify individuals in the same way that ISBNs and DOIs identify books and articles. Thus the risk of confusing your identity with another researcher with the same name is eliminated. **The ORCID website** provides researchers with a page where your comprehensive research activity can be stored.

Springer Nature collaborates with the ORCID organization to ensure that your research contributions (as authors and peer reviewers) are correctly attributed to you. Learn more at https://www.springernature.com/gp/researchers/orcid

Other data requests
We strongly encourage the deposition of your full microscopy image data sets in the Image Data Resource: https://idr.openmicroscopy.org/about
**Code availability and citation**

Thank you for making your custom code available via
Github. Upon publication, Nature Portfolio journals consider it best practice to release custom computer code in a way that allows readers to repeat the published results. Code should be deposited in a DOI-minting repository such as Zenodo, Gigantum or Code Ocean and cited in the reference list following the guidelines described in our policy pages (see link below). Authors are encouraged to manage subsequent code versions and to use a license approved by the open source initiative.

See here for more information about our code availability policies: https://www.nature.com/nature-portfolio/editorial-policies/reporting-standards#availability-of-computer-code

Data presentation

Please state in the figure legends how many times each experiment was repeated independently with similar results. This is needed for all experiments, but is particularly important wherever results from representative experiments (such as micrographs) are shown. If space in the legends is limiting, this information can be included in a section titled "Statistics and Reproducibility" in the methods section.
