# Supplementary material for: Super-resolved trajectory-derived nanoclustering analysis using spatiotemporal indexing
Source: Nat Commun. 2023 Jun 8;14:3353. doi: 10.1038/s41467-023-38866-y (PMC10250379; doi:10.1038/s41467-023-38866-y)
Supplement: Supplementary file 2 — Supplementary Figures [file 41467_2023_38866_MOESM2_ESM.docx]

**Super-resolved trajectory-derived nanoclustering analysis using spatio-temporal indexing**

Tristan P. Wallis^1,#^, Anmin Jiang^1^, Kyle Young^2^, Huiyi Hou^1^, Kye Kudo^1^, Alex McCann^1^, Nela Durisic^3^, Merja Joensuu^1^, Dietmar Oelz^2^, Hien Nguyen^2^, Rachel S. Gormal^1,#^, and Frédéric A. Meunier^1,4,#^

^1^Clem Jones Centre for Ageing Dementia Research, Queensland Brain Institute, The University of Queensland, Brisbane, QLD 4072, Australia.

^2^School of Mathematics and Physics, The University of Queensland, Brisbane, QLD 4072, Australia.

^3^Queensland Brain Institute, The University of Queensland, Brisbane, QLD 4072, Australia.

^4^School of Biomedical Sciences, The University of Queensland, Brisbane, QLD 4072, Australia.

# Corresponding authors: Email: [f.meunier@uq.edu.au](mailto:f.meunier@uq.edu.au), [r.gormal@uq.edu.au](mailto:r.gormal@uq.edu.au) and [t.wallis@uq.edu.au](mailto:t.wallis@uq.edu.au)

**Supplementary Figures 1 - 10**

**
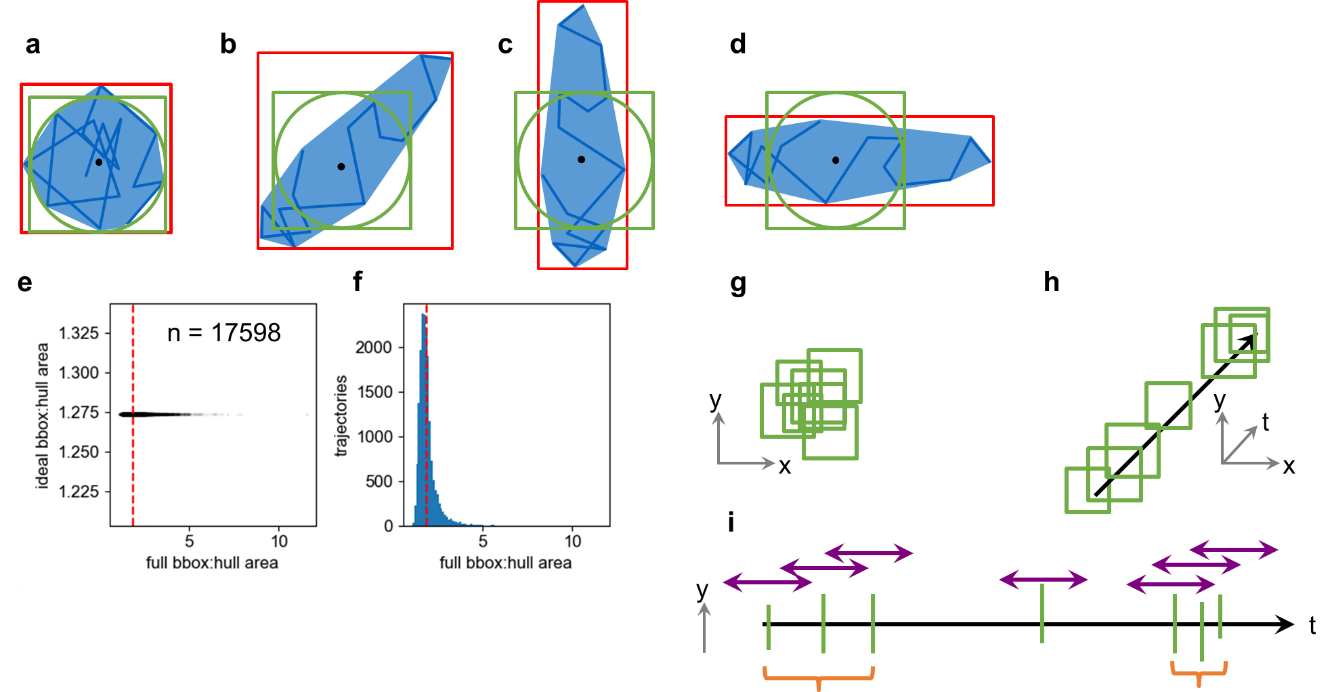
**

**Supplementary Fig. 1** | Use of ideal regular bounding regions for spatio-temporal indexing. Spatial indexing using R-tree requires use of rectangular bounding boxes rather than the irregular convex hull containing each trajectory. **a-d**, the convex hull (light blue) of a trajectory (dark blue) is encompassed by a rectangular bounding box (red) which describes the full extent of the trajectory. The area of the convex hull is used to compute a circle of the same area (green), which is encompassed by an ideal bounding square (green). For an approximately circular convex hull (**a**) the full bounding box and ideal bounding square are similar. For an elliptical convex hull (**b-d**) the area of the full bounding box is related to the angular offset of the ellipse axes to the x and y axes. The area of the ideal bounding square is invariant. **e**, Comparison of the full bounding box and ideal bounding square for trajectories of syntaxin1A, as described in the main text. For each trajectory, the ratio of the ideal bounding square area to the convex hull area is plotted on the y axis, and the ratio of the full bounding box area to the convex hull area is plotted on the x axis. Full bounding boxes tend to overreport the area of each trajectory The red dotted line represents the average ratio of the full bounding box to the convex hull (1.892 ± 0.0037), which is higher than the ratio of the ideal bounding square to the convex hull 2π2/πr2 = 1.273. **f,** histogram of distribution of the ratio of the full bounding box area to the convex hull area. **g-i**, use of ideal bounding squares to establish spatio-temporal clustering. **g**, overlapping ideal bounding squares (green boxes) represent spatial clustering of the parent trajectories. **h,i,** progressive rotation of the figure to show temporal distribution of the bounding squares based on the temporal centroid of each trajectory. The purple lines above each rotated bounding square represent the time window (t) as described in the text. For t = 20s, any two bounding squares within 10s are considered as temporally overlapping. Bounding squares highlighted with an orange bracket represent spatio-temporal clusters whose component trajectories have overlapping time windows. The isolated bounding square in the middle of the temporal line does not overlap with any others and is not considered to be part of a spatio-temporal cluster. For large values of t, all time windows will overlap, and the data will represent a single spatio-temporal cluster. Source data are provided as a Source Data file.


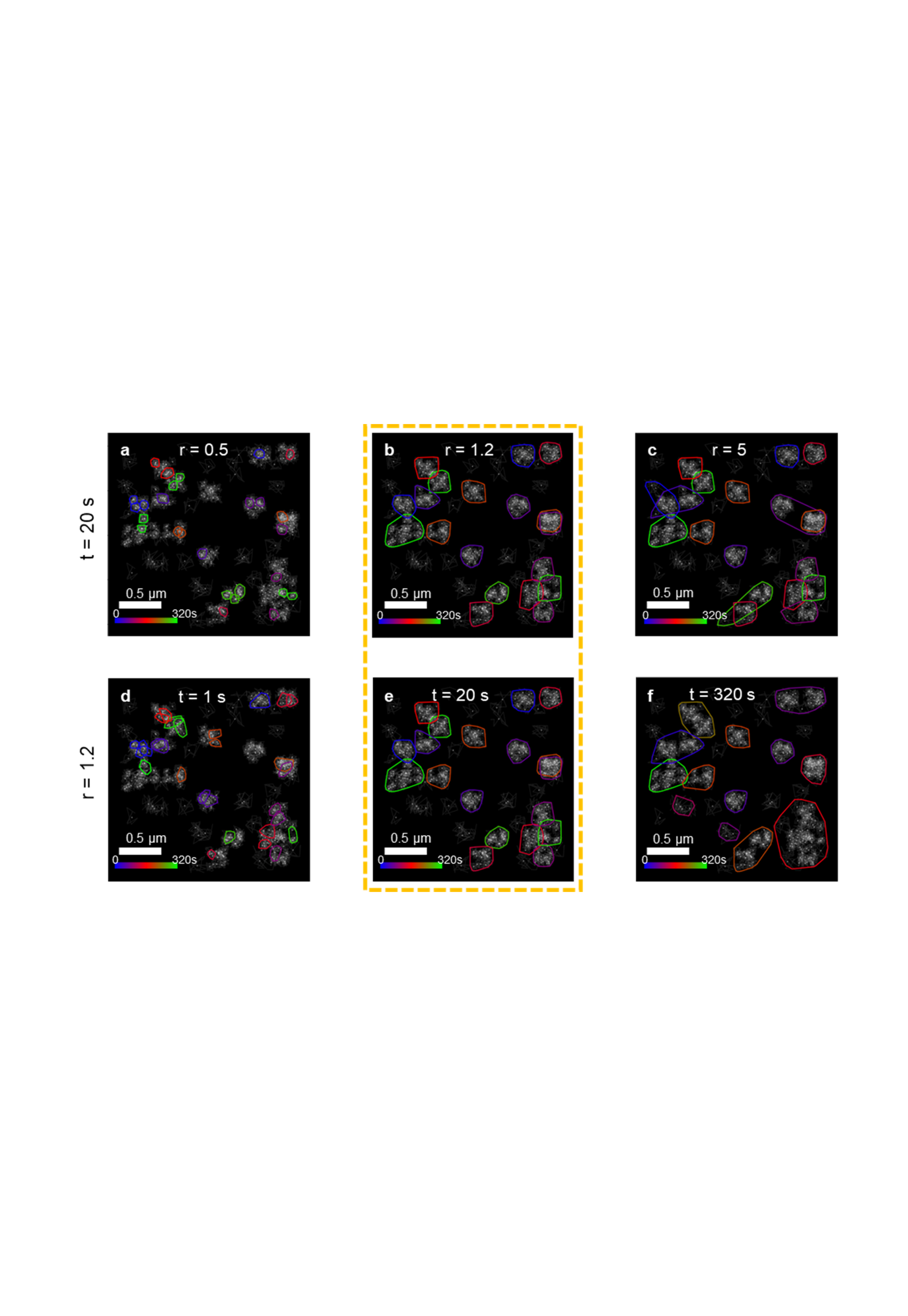


**Supplementary Fig. 2 |** Bounding box radius and time window affect spatio-temporal clustering. *In silico* random walk trajectory data consisting of 20 equivalently sized spatio-temporal clusters where each cluster contains 20 trajectories distributed within 10 s. Clusters are randomly distributed within a 320 s “acquisition” window. The spatial centroid of each trajectory is represented as a dark dot. **a, b, c:** Spatio-temporal clustering was performed with a time window of 20 s, with each trajectory’s bounding box radius multiplied by the indicated factor *r*. **d, e, f:** Spatio-temporal clustering was performed with a bounding box radius factor of 1.2, and an indicated time window *t*. A cluster is defined as 3 or more proximal centroids. Cluster boundaries represent the extent of the detections associated with clustered trajectories, and are colored according to the average detection time as indicated by the color bar. Dotted box represents clustering obtained with r = 1.2, t= 20 s, most representative of the ground truth of the input data. Source data are provided as a Source Data file.


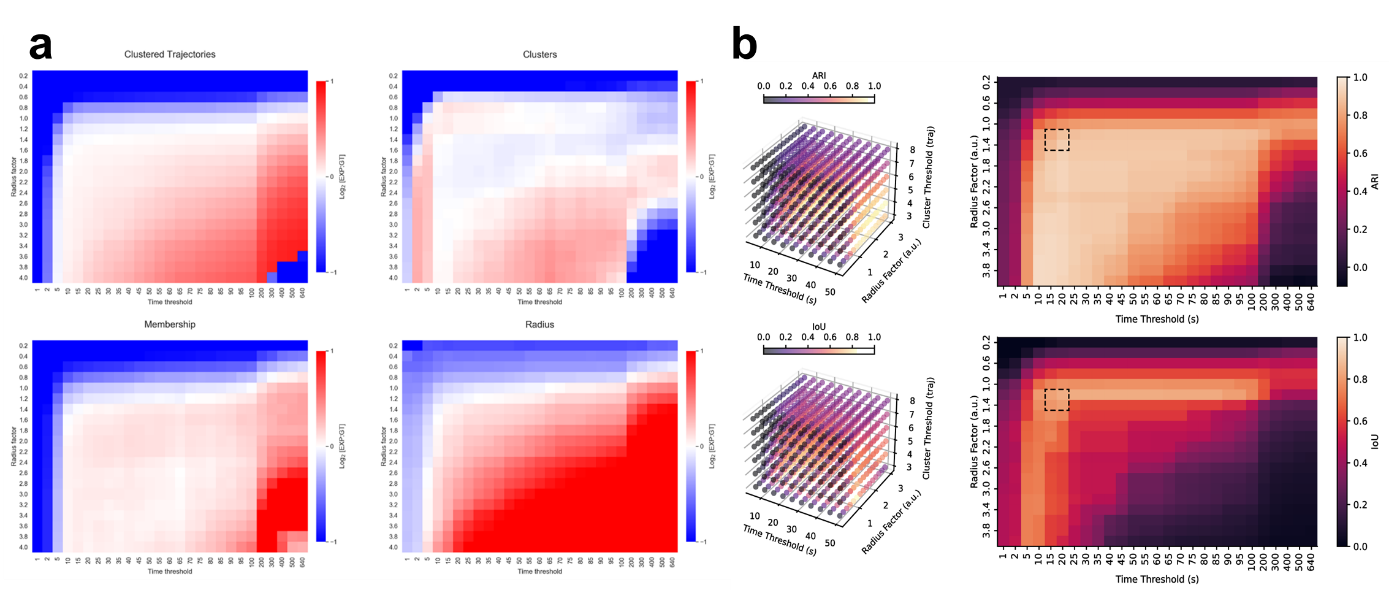


**Supplementary Fig. 3 |** Effect of bounding box radius and time window on spatio-temporal clustering metrics. *In silico* random walk trajectory data consisting of 1095 trajectories in 140 spatio-temporally unique clusters with 7.82 ± 0.16 trajectories per cluster, with cluster radii of 74.86 ± 5.29 nm. The data also contained a background of 1000 randomly spatio-temporally distributed unclustered trajectories. Clusters are randomly distributed within a 320 s “acquisition” window. **a,** For a given metric, each pixel represents the log_2_ ratio of the experimental observed (EXP) value to the ground truth (GT). Ratios < -1 and > 1 are displayed as 1 and -1 respectively. **b,** Adjusted Rand Index (ARI) and Intersection over Union (IoU) show a similar “inflection” point to that shown in Fig. 1o. Source data are provided as a Source Data file.

**
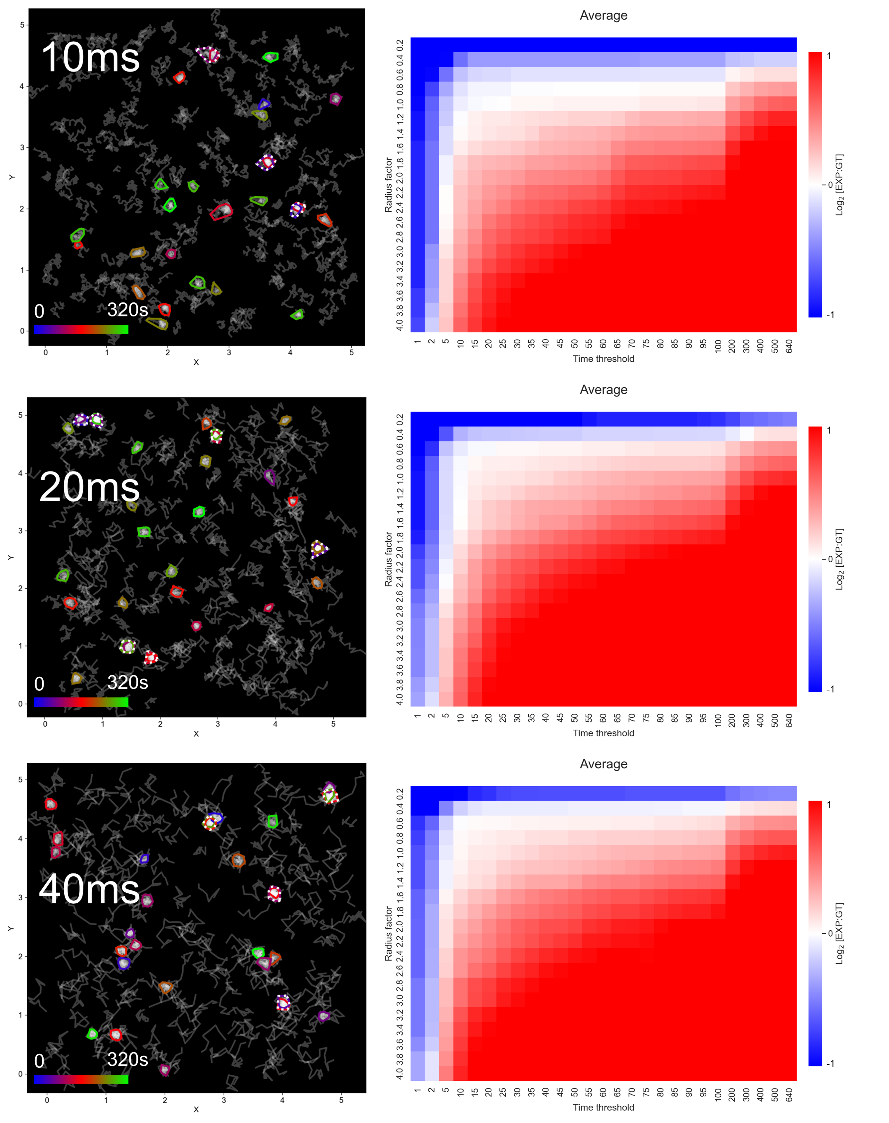
**

**Supplementary Fig. 4 |** Applicability of NASTIC parameters to different SMLM data geometries. Synthetic trajectory data was generated such that each dataset contained equivalent numbers of similarly sized clusters randomly distributed within a 320 s “acquisition” window. with approximately 6 trajectories per cluster. The number of background randomly spatio-temporally distributed unclustered trajectories was the same for each dataset. Datasets differed according to simulated acquisition frame time, trajectory length and average trajectory segment step length such that across frame times, the average area encompassed by trajectories was similar. This simulates acquiring data on the same molecules using instrumentation with different acquisition rates. NASTIC was performed using a matrix of radius factors and time thresholds. **Left panels**, clusters returned using ***r*** = 1.2, ***t*** = 20 s. Dotted lines represent “hotspots” where multiple clusters were detected in the same spatial area but at different times. **Right panels**, heatmaps of averaged metrics (cluster number, cluster radius, trajectories per cluster and number of clustered trajectories). Each pixel represents the average log_2_ ratio of the experimental observed (EXP) value for a given *r/t* pair to the ground truth (GT). Pale regions indicate *r/t* pairs which return cluster metrics close to the ground truth. **Upper panels**, 10 ms frame time (100 Hz acquisition), 8-60 steps, average step length 25 nm. **Middle panels**, 20 ms frame time (50 Hz acquisition), 8-30 steps, average step length 50 nm. **Bottom panels**, 40 ms frame time (25 Hz acquisition), 8-16 steps, average step length 100 nm. Source data are provided as a Source Data file.


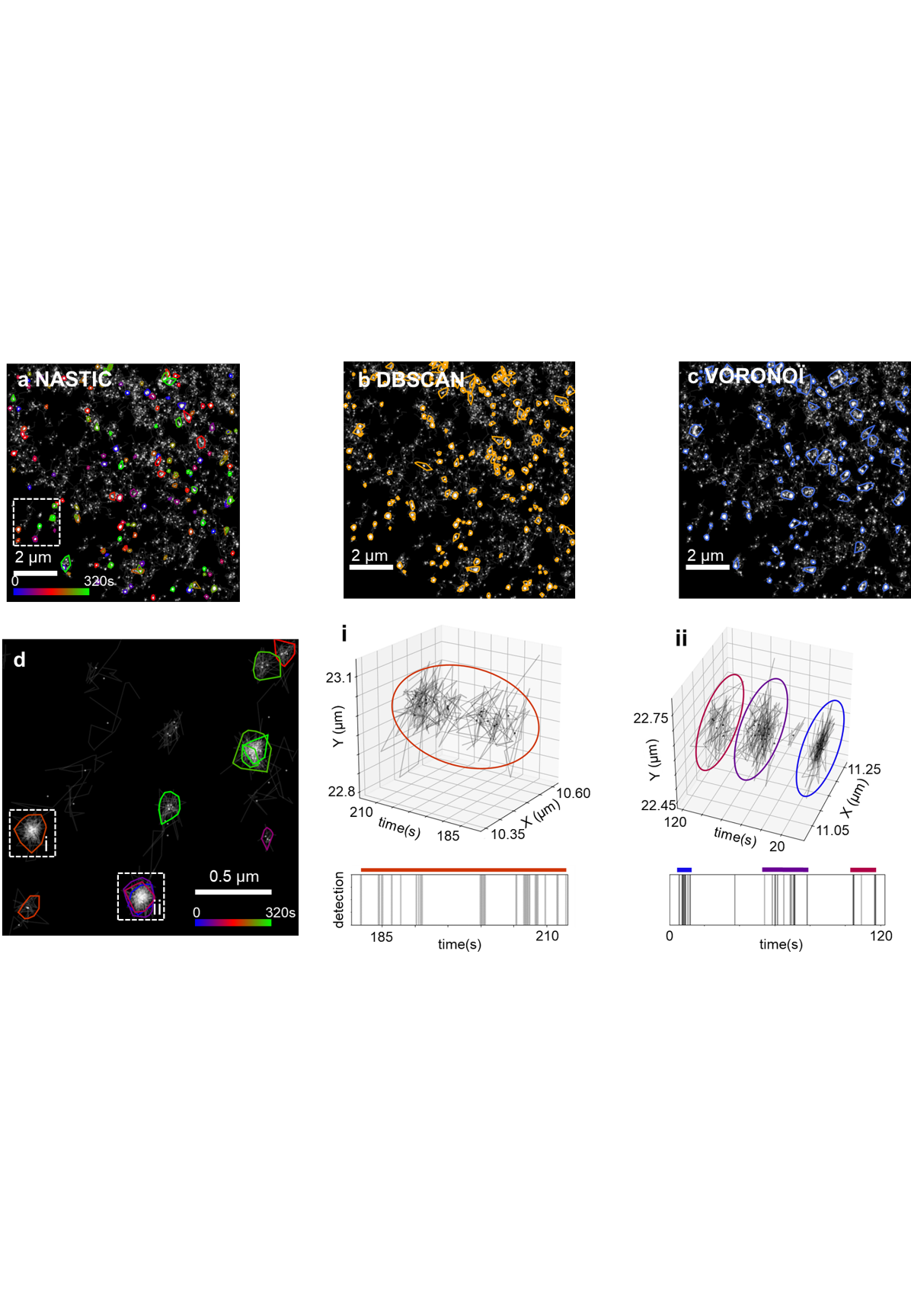


**Supplementary Fig. 5 |** Resolution of spatio-temporal clustering in live-cell molecular trajectory data. Sx1a-mEos2 sptPALM data acquired at 50 Hz over 320 s. Clustering based on **a,** NASTIC using ***r*** = 1.2, ***t*** = 20 s, **b,** DBSCAN using **ε =** 0.055 μm and MinPts = 3 and **c,** Voronoï tessellation using tile threshold 0.015 μm^2^. **d,** Magnification of the region indicated by the dotted box in **a**. Cluster boundaries represent the extent of the detections associated with clustered trajectories, and are colored according to the average detection time. Insets highlight different classes of clustering further visualized by 3D (x, y, t) projections of highlighted clusters and the associated detection times: **i,** single spatio-temporal cluster; **ii,** spatially overlapping clusters resolved in time. Source data are provided as a Source Data file.


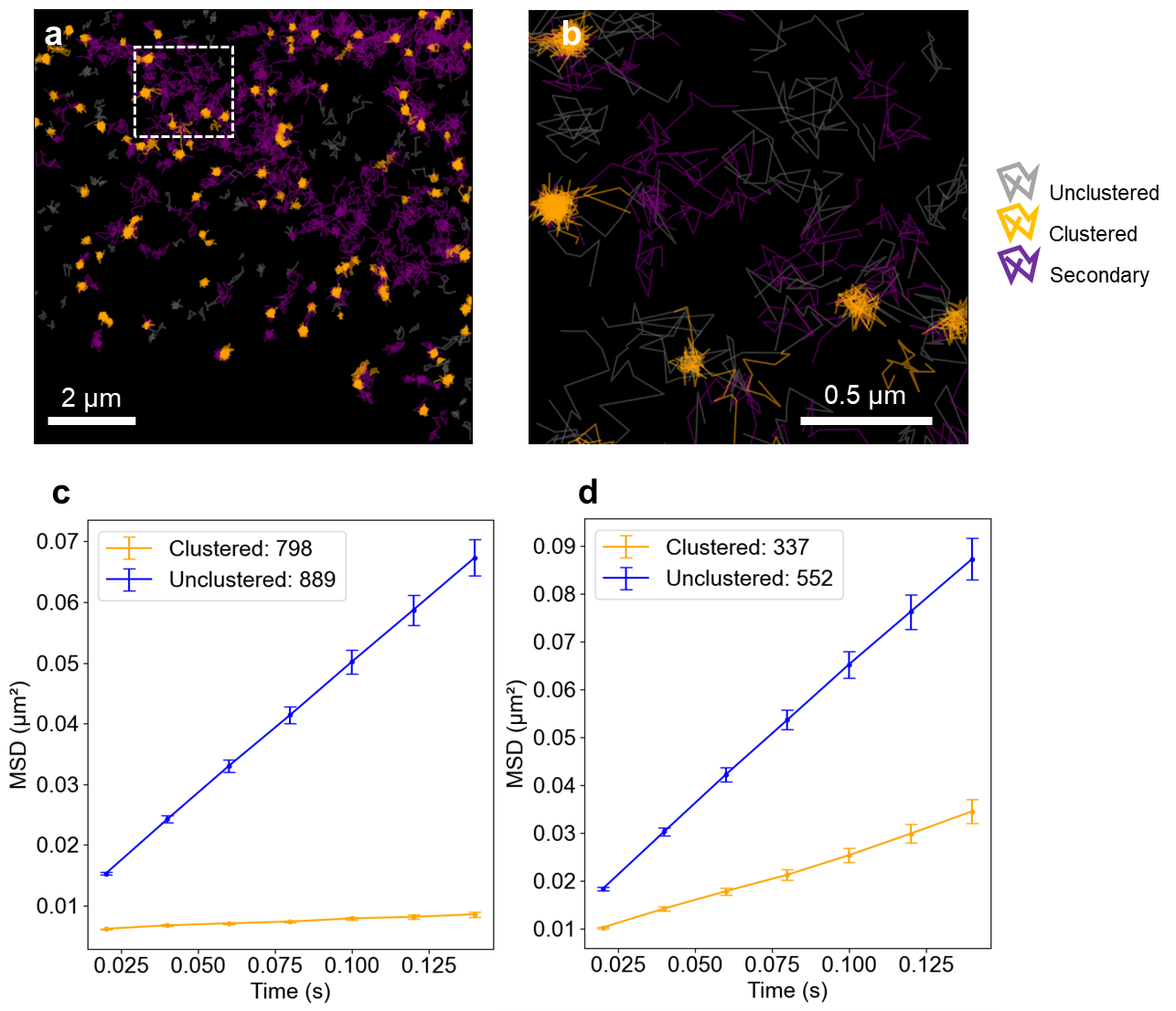


**Supplementary Fig. 6 |** Identification of time resolved (primary) and non-time resolved (secondary) clusters reveals clustering hotspots. Sx1a-mEos2 sptPALM data acquired at 50 Hz over 320 s. **a,** Analysis using spatio-temporal indexing using ***r*** = 1.2, ***t*** = 20s. Unclustered trajectories are shown in grey, spatially discrete clustered trajectories are shown in orange, trajectories belonging to spatially overlapping clusters are shown in green. Secondary analysis of the unclustered trajectories from the primary analysis, using ***r*** = 1.2, ***t*** = 640. Trajectories in secondary clusters are shown in purple. Dotted box indicates the area enlarged in **b**. **c** and **d,** Mean square displacement (MSD) curves of clustered and unclustered trajectories from the primary analysis and secondary analysis respectively. Each point represents the average MSD of the indicated number of trajectories. Error bars indicate the standard error of the mean (SEM). Source data are provided as a Source Data file.


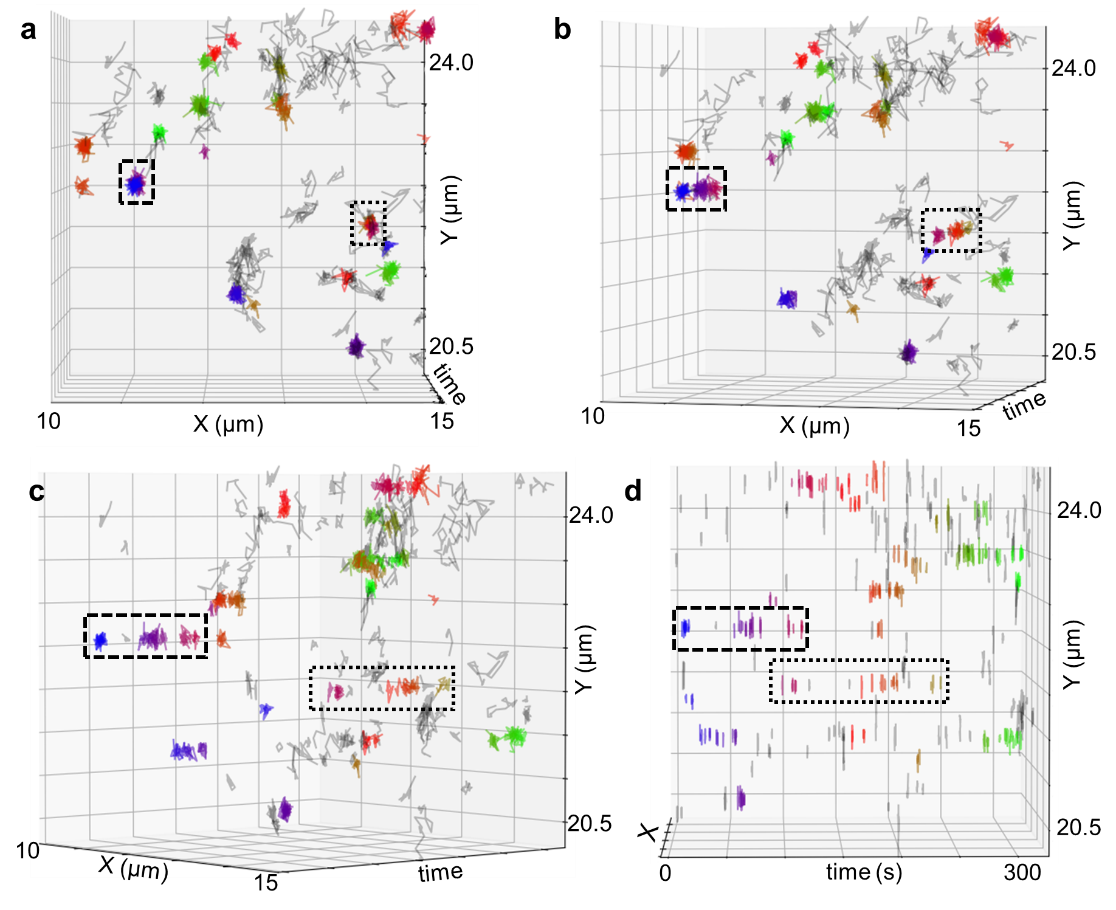


**Supplementary Fig. 7 | a-d,** Progressive rotation around the y-axis of the 3D [x,y,t] projection of spatio-temporal Sx1a-mEos2 trajectory data, with clustered trajectories indicated in color. Dotted and dashed boxes show representative single spatial clusters which resolve into multiple spatio-temporal clusters (hotspots). The dashed box corresponds to the spatio-temporal hotspot observed in Fig. 3c,f. Source data are provided as a Source Data file.


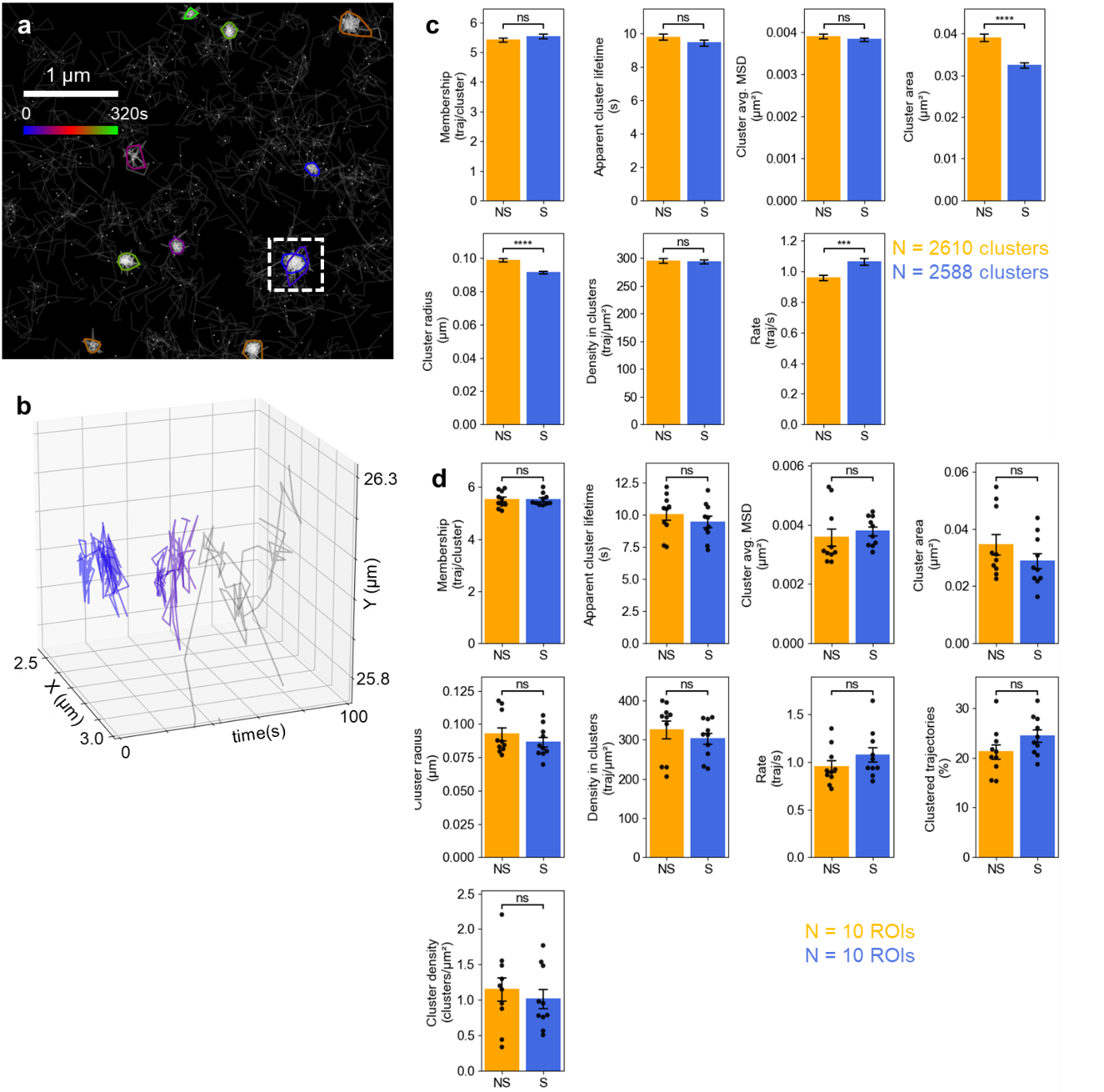


**Supplementary Fig. 8 |** Difference in cluster metrics in response to stimulated exocytosis. Munc18-1-mEos2 sptPALM data acquired from unstimulated PC12 cells (N=9) and PC12 cells stimulated with 2 mM BaCl_2 (_N=9). **a,** Representative image of clusters identified using spatio-temporal indexing with ***r*** = 1.2, ***t*** = 20 s. Dotted box highlights an overlapping cluster hotspot **b,** 3D (x, y, t) projection of highlighted hotspot clusters. **c,** Comparison of indicated cluster metrics of N = 2610 pooled clusters from unstimulated cells and N = 2588 clusters from stimulated cells. The significance of the difference between conditions was determined by unpaired two-tailed *t*-test (ns = no significance, *** = p < 0.001, **** = p < 0.0001). NS = no stimulation, S = stimulation with 2 mM BaCl_2_. Error bars represent standard error of the mean (SEM). **d,** Comparison of indicated average cluster metrics. One ROI was analysed from each of 10 biological samples in a single experiment. The significance of the difference between conditions was determined by unpaired two-tailed *t*-test (ns = no significance). Error bars represent SEM. Source data are provided as a Source Data file.


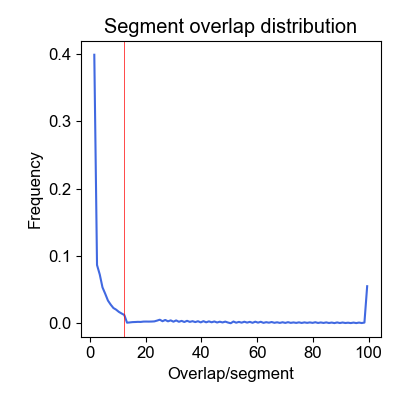


**Supplementary Fig. 9 |** Distribution of segment overlap. Sx1a-GFP imaged by uPAINT using Atto-647 labelled anti-GFP nanobodies in PC12 cells. The vertical red line corresponds to the average segment overlap. Segments with overlap greater than this value are considered potentially clustered. Source data are provided as a Source Data file.


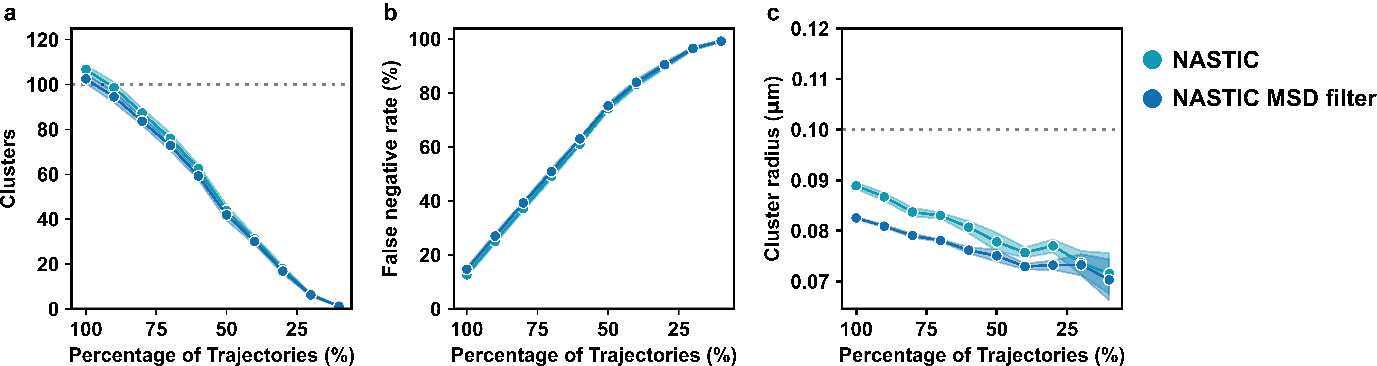


**Supplementary Fig. 10 |** Global reduction of trajectory density reduces cluster detectability of NASTIC. Results from computational simulations in which the total number of trajectories (i.e., background density of 20 trajectories / µm^2^ and 100 clusters, each containing 3 to 8 trajectories, within a 100 µm^2^ region) was iteratively reduced via sampling a given percentage. Simulated trajectories were 8 to 30 frames in length. **a,** number of clusters detected **b**, false negative rate and **c,** radius of identified clusters are altered as the trajectory density is reduced (*n* = 10 simulations). Data represented as $\bar{x}$ ± SEM; ground-truth values represented as grey dotted lines. Source data are provided as a Source Data file.
